# Experiences, impact, and enablers of involving young people and family caregivers in developing reporting guidelines for paediatric randomised trials: a case study

**DOI:** 10.1186/s40900-025-00751-x

**Published:** 2025-07-01

**Authors:** Ami Baba, Maureen Smith, Jennifer Preston, Begonya Nafria Escalera, Segolene Gaillard, Pamela Dicks, Matthew Prebeg, Amanda Doherty-Kirby, Kimberly Courtney, Lynn Mendonza, Tanya Chute Nagy, Lotty Hooft, Martin Offringa

**Affiliations:** 1 Child Health Evaluative Sciences, The Hospital for Sick Children Research Institute, Toronto, ON Canada; 2 INFORM RARE Research Network, Ottawa, ON Canada; 3 National Institute for Health and Social Care Research (NIHR), Alder Hey Clinical Research Facility, Liverpool, UK; 4 Institut de Recerca Sant Joan de Déu, Barcelona, Spain; 5 Universitat Oberta de Catalunya, Barcelona, Spain; 6 CIC Inserm, Kids France, CHU-Lyon, Lyon, Bron, 1407, 69677 France; 7 Laboratoire de biométrie et biologie évolutive, Université Lyon 1, CNRS UMR 5558, Villeurbanne, 69622 France; 8 NHS NRS Children’s Research Network, Royal Aberdeen Children’s Hospital, Aberdeen, Scotland, UK; 9 Centre for Addiction and Mental Health, Toronto, ON Canada; 10 SPIRIT | CONSORT-Children and Adolescents Family Caregiver Advisory Group, Toronto, ON Canada; 11 Ontario Child Health Support Unit, CHEO Research Institute, Ottawa, ON Canada; 12 Canadian PKU and Allied Disorders, Toronto, ON Canada; 13 Cochrane Netherlands, Julius Center for Health Sciences and Primary Care, University Medical Center Utrecht, Utrecht University, Utrecht, The Netherlands

**Keywords:** Reporting guideline, Clinical trial, Randomised controlled trial, Paediatrics, Children, Adolescents, Youth involvement, Family involvement, Patient and public involvement

## Abstract

**Background:**

Patient and public involvement (PPI) is increasingly recognized as important, yet no guidance exists on integrating young people and family caregiver perspectives in the development of research reporting guidelines. We developed two paediatric-specific extensions with young people (ages 10–24 years) and family caregivers (YPFC) for the Standard Protocol Items: Recommendations for Interventional Trials (SPIRIT) and Consolidated Standards of Reporting Trials (CONSORT) reporting guidelines: SPIRIT-Children and Adolescents 2025 (SPIRIT-C) and CONSORT-Children and Adolescents 2025 (CONSORT-C). This case study describes how we involved YPFC in the development of SPIRIT-C and CONSORT-C and identified enablers of impactful PPI.

**Main text:**

We formed a Youth Advisory Group (ages 13–19 years) and a Family Caregiver Advisory Group. A miniseries of two Young Person Reporting Guideline workshops aimed at generating randomised controlled trials (RCT) reporting items were conducted virtually in Canada, England, France, Scotland, and Spain, engaging 42 young people (ages 10–21 years). Young people (ages 19–24 years) and family caregivers participated as panellists in an international Delphi study. Family caregiver advisors actively contributed to the Consensus Meeting and to the writing process of the guidelines’ Explanation and Elaboration (E&E) documents. After each project stage, YPFC feedback was collected. PPI impact was defined as tangible changes, learnings, and outcomes, both positive and negative, to the guideline development process and the final guidelines resulting from YPFC co-development. YPFC found their involvement in the project a valuable experience. Their contributions to key project stages, such as the Delphi study, Consensus Meeting, and the development of the E&E documents impacted the final guidelines and E&E documents, with the inclusion of four new youth generated reporting items. Feedback throughout the project informed six “enablers” for productive partnerships in reporting guideline development: (1) designated point person, (2) tailored training, (3) access to project materials, (4) clear expectations on time commitment and compensation, (5) structured check-in sessions, and (6) demonstrated openness to feedback.

**Conclusion:**

With careful preparation, investing in impactful PPI enablers, YPFC can meaningfully contribute to the development of research reporting guidelines, improve final deliverables, and ultimately shape research that reflects their perspectives.

**Supplementary Information:**

The online version contains supplementary material available at 10.1186/s40900-025-00751-x.

## Abstract (plain language summary)

Young people and family caregivers (YPFC) are end-users of paediatric research reports. To ensure that items of importance to them are reported, they should have a say in what key items of a study should be reported. While there is increased interest in involving YPFC in health research, guidance on how best to involve young people ages 10–24 years and family caregivers in the development of research reporting guidelines does not exist. Recently, recommendations on how patient and public partners can be meaningfully involved in research reporting guideline development were published, though these recommendations were informed by the experiences of adult partners. This study is the first to involve YPFC in the development of reporting guidelines for paediatric randomised controlled trials (RCTs). It describes the experience of this involvement from multiple perspectives and evaluates YPFC’s impact on the final guidelines. YPFCs contributed to the generation of candidate reporting items, a three-round Delphi study, Consensus Meeting, and writing/review of the Explanation and Elaboration documents. Using formal evaluations after each project stage, we identified six enablers for their effective involvement in future projects that aim to add value to child health research by integrating YPFCs perspectives. Investing in enablers for effective YPFC involvement may improve research practice and yield increased relevance and utility of child health research deliverables and outcomes for end-users and add value to research reflecting YPFC perspectives.

**Supplementary Information:**

The online version contains supplementary material available at 10.1186/s40900-025-00751-x.

## Introduction

Well-designed and appropriately conducted randomised controlled trials (RCTs) provide reliable evidence needed to evaluate the efficacy and effectiveness of interventions and improving health research outcomes in children and young people. Suboptimal reporting in paediatric RCTs is pervasive, with key details on trial interventions, outcomes, consent/assent procedures, and sample size calculations often omitted [1]. This contributes to ongoing, yet avoidable research waste [2]. Inadequate reporting prevents the reader’s ability to properly interpret the methods and findings of studies [3], which has consequences for healthcare decision-making and evidence synthesis [4].

Despite the availability of multiple reporting guidelines focused on different trial designs, conditions, interventions, and outcomes [5], no reporting guideline that factors in considerations unique to paediatric RCTs exist. Given the perceived suboptimal reporting in paediatric RCT protocols and reports [1, 6, 7], multiple calls have expressed the need for harmonised reporting guidelines specific to paediatric RCTs [1, 4, 8–10]. To date, only four reporting guidelines about paediatric research have been published [4]. However, none are specific to paediatric RCTs nor have they integrated the perspectives of young people and family caregivers (YPFC) in their development.

YPFC are end-users of paediatric research reports. True to Article 12 of the United Nations Convention on the Rights of the Child (UNCRC) [11], they should have a say in what key items of a study should be reported. Critically, reporting guidelines that consider what is important to young people and families enable informed healthcare decisions. Outside of paediatrics, the importance of PPI in the development of reporting guidelines is increasingly recognized [12–15]. Although adding the perspectives and lived experience of patient and public partners increases the understanding of the priorities of end-users, PPI is not common practice in reporting guideline development. Studies show that PPI in research has many benefits, such as increasing the relevance and utility of research findings [10, 16–18]. When it comes to research reporting, partnering with YPFC may yield reporting guidelines that are reflective of their priorities, thereby increasing the value of research and reducing research waste [2, 10].

Recently, Elsman et al. published a “blueprint” paper with 17 recommendations on how to meaningfully involve patient and public partners in the development of research reporting guidelines [14]. Manyara et al. detailed six recommendations regarding involvement of patient and public partners in reporting guideline development for surrogate outcomes in trials [15]. Both emphasized the importance of having a clear partner involvement plan, budget for compensation and reimbursements, proper onboarding/training, remaining flexible, and reflecting on the impact of PPI. However, these recommendations were developed without involvement of YPFC.

To improve reporting in paediatric RCT protocols and reports, we developed an extension to the Standard Protocol Items: Recommendations for Interventional Trials (SPIRIT) [19] and Consolidated Standards of Reporting Trials (CONSORT) [20] reporting guidelines that are specific to paediatric RCTs: *SPIRIT-Children and Adolescents* (SPIRIT-C) 2025, and *CONSORT-Children and Adolescents* (CONSORT-C) 2025 [21, 22]. We involved YPFC in all project stages to develop guidelines that are relevant and useful to end-users. YPFC were included in the generation of candidate reporting items, the Delphi study, Consensus Meeting, and writing/review of the Explanation and Elaboration (E&E) documents.

With no existing guidance on how best to involve YPFC in the development of research reporting guidelines, the aim of this case study was to prospectively (a) evaluate young people and family caregivers’ involvement and experience in the development of paediatric research reporting guidelines, and (b) assess the impact resulting from their contributions and involvement. Based on YPFC’s feedback, we set out to identify the key enablers of meaningful and impactful involvement.

## Methods

Guidelines or best practices specific to involving young people and families in the development of reporting guidelines do not exist. Therefore, we implemented the “blueprint” recommendations of Elsman et al. [14] (eFigure 1) and the recommendations by Manyara et al. [15] on effective ways to involve adult patient and public members in the development of reporting guidelines. Details on our planning, considerations, expertise, and formation of the International Youth Involvement Steering Committee are documented in Additional file 1, Section B. The International Youth Involvement Steering Committee planned and guided the involvement of YPFC throughout the project and comprised youth facilitator experts from the European Young Person Advisory Group Network (eYPAGnet) (JP, BNE, SG, PD) and core project team members (MS, AB, MO).

We integrated lessons learned and recommendations from our previous experiences of working with young people and families in methodological work or throughout the research process [23, 24]. This included adopting a responsive methodology, which embodies relational empowerment, fluidity, and flexibility [24]. We also strived to apply recommendations to create a safe, open, and inclusive environment, and to recognize each partner’s unique experiences, knowledge, and background as expertise [23].

### YPFC involvement

#### Young people

In context of the SPIRIT-C 2025 and CONSORT-C 2025, and as defined in both statement papers [21, 22], the term “paediatric” includes child and adolescent health. Both guidelines pertain to trials that evaluate any intervention(s) (i.e., pharmacological and non-pharmacological, including public and population health) in newborns, infants, children, and adolescents (ages 0–19 years); this age range is based on the UNCRC and the World Health Organization (WHO) definitions [25–28].

**Fig. 1:**
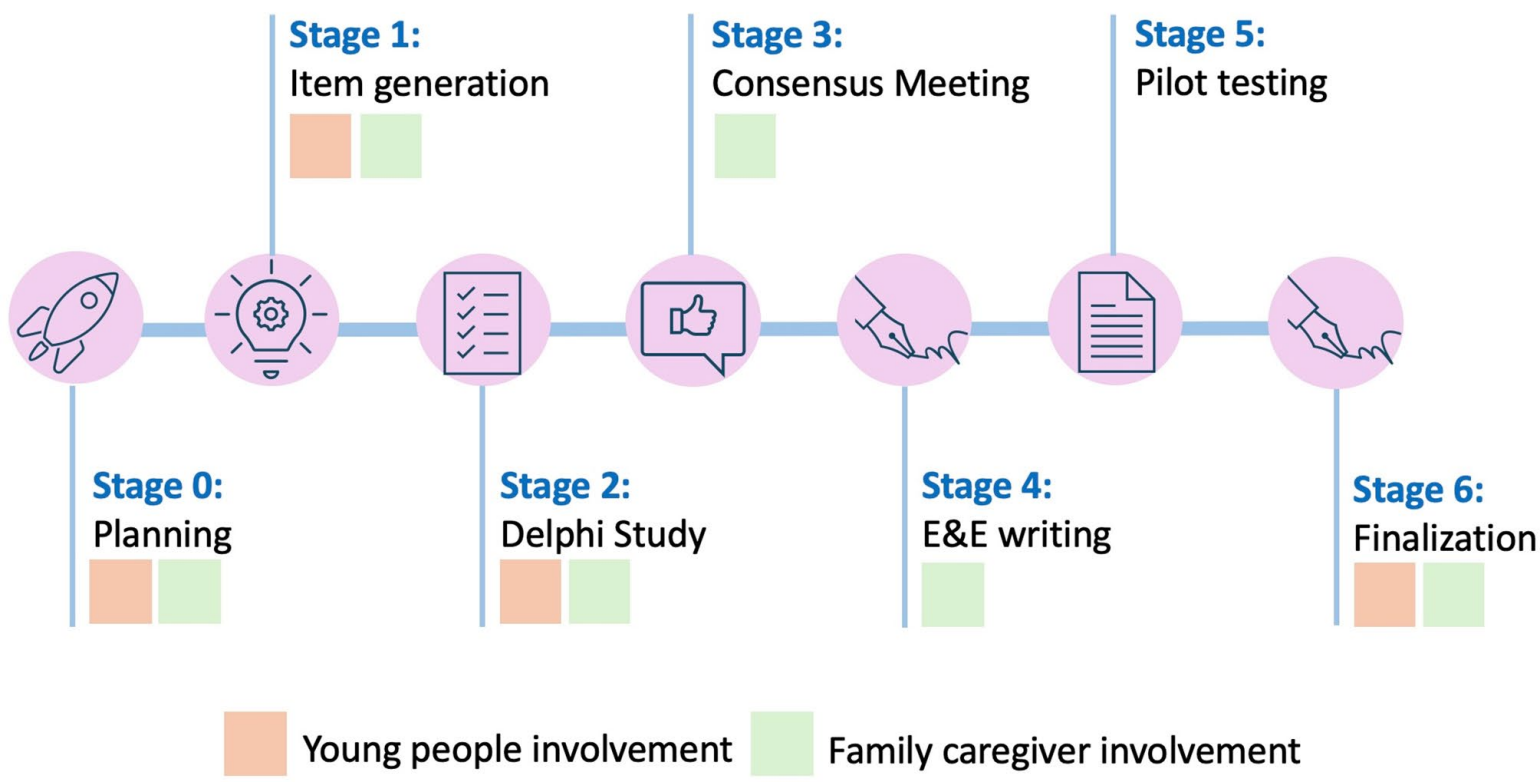
Involvement of young people and family caregivers throughout project stages

The involvement of young people in the development of SPIRIT-C and CONSORT-C, and in the various project stages, are described in detail in Additional file 1, Section B, 1–3. We summarise their involvement thoughout the project in Fig. 1, and provide details in Table 1.

**Table 1:** Involvement of young people (ages 10–24 years), family caregivers, and paediatric trial experts in the development of SPIRIT | CONSORT-Children & adolescents 2025

Project Stage	Involvement of key partner groups	
	Young people (ages 10–24 years)	Family caregivers
0. Planning	Formation of Youth Advisory Group (*n* = 6, ages 13–19 years) Development of Young Person Reporting Guideline (YPRG) workshop materials (i.e., workshop design, content, approach, recruitment materials)	Formation of Family Caregiver Advisory Group (*n* = 5) Development of onboarding/training session for Delphi study and identification of potential Delphi panellists (older youth (ages 19–24), other family caregivers)
1. Generation of candidate items	Two virtual YPRG Workshops with 42 young people ages 10–21 years	See Stage #2, below
2. Delphi Study	Three-round, online survey for youth ages 19–24 years (*n* = 4)	Vote on inclusion/exclusion of candidate reporting items in a three-round, online survey; all panellists had the opportunity to suggest new items during Round 1
3. Consensus Meeting	Contributions generated at Stage #1 were discussed at the meeting, with representation by youth facilitator experts	Discussions and anonymous voting during a virtual meeting
4. E&E writing*	Excluded by design	Writing/review using an online shared document
5. Pilot testing	Excluded by design	Excluded by design
6. Finalization	With their permission, acknowledged on manuscripts Youth Advisory Group meeting – brainstorm on youth appropriate knowledge translation materials, developed with youth advisors	Critical review/revision of manuscripts by those who qualified for co-authorship as per International Committee of Medical Journal Editors (ICMJE) criteria Family Caregiver Advisory Group meeting – brainstorm on knowledge translation materials, developed with family advisors

*Explanation and Elaboration papers

To involve a wide range of current and recent paediatric perspectives, we decided to involve young people (ages 10–24 years) who either (a) have lived experience of participating in a paediatric RCTs; (b) read research and use trial results to make healthcare decisions for themselves; or (c) have experience collaborating with researchers on a research team (e.g., through participation in YPAGs) throughout the project. The included age range is based on the WHO definition of “young people,” which comprises individuals ages 10–24 years [25], and the age range of the five international Young Person’s Advisory Groups (YPAG’s) membership (youngest age of 10 years). We formed a Youth Advisory Group (YAG) with six young people (ages 13–19 years; average: 16.6 years) with relevant experiences and backgrounds, of which four were female and two were male. Youth advisors resided in the Canadian provinces of Alberta (*n* = 2), British Columbia (*n* = 2), Ontario (*n* = 1), and Quebec (*n* = 1). We offered young people flexibility in how they could be involved with the project depending on their age and desired level of involvement. For example, youth advisors had a choice to attend the Young Person Reporting Guideline (YPRG) workshop if it was of interest to them.

#### Family caregivers

Methods of involving family caregivers were modelled after the PPI strategy implemented in the development of the PRISMA-COSMIN for Outcome Measurement Instruments (OMIs) guideline; specifically, we followed the 17 blueprint recommendations (eFigure 1) [14]. In brief, we formed a Family Caregiver Advisory Group (FCAG) comprising family caregivers (e.g., parents, guardians) of children who participate(d) in a paediatric trial, and/or those who read research to access trial results to help in healthcare decision-making for their child/children. The FCAG comprised five parents (4 females, 1 male) residing in the Canadian provinces of Ontario (*n* = 3), Quebec (*n* = 1), and Prince Edward Island (*n* = 1). Four caregivers have child(ren) who participate(d) in a paediatric trial. All reported reading trial results to inform healthcare decision making for their child. Family caregiver advisors were involved throughout the project to advise on content, material, and conduct of various activities (e.g., Delphi study). They also advised on knowledge translation and dissemination strategies. In addition to being part of the FCAG, advisors had the opportunity to contribute to subsequent project stages, including the Delphi study, Consensus Meeting, and Explanation and the Elaboration (E&E) writing process. Family caregivers with similar experiences and backgrounds who were not in the FCAG were also later identified to contribute as a Delphi panellist.

Family caregivers were involved throughout the entire project (Table 1) and were offered flexibility in terms of their time and contribution commitment, as described in Additional file 1, Section C, 1–4.

YPFC were recognized for their time and contributions as detailed in eTable 1, Additional file 1.

### Involvement experience and impact

Throughout the development of SPIRIT-C 2025 and CONSORT-C 2025, we evaluated the experiences of the involved YPFC. In addition to documenting these experiences, we wanted to capture the impact of YPFC’s involvement on the development process and final reporting guidelines. Interest in assessing and measuring PPI impact on research is growing [29, 30]. However, there is no standard definition of such impact. Based on three published definitions, we define “PPI impact” as both positive and negative tangible changes, learnings, and outcomes, on both the development process and to the final guidelines [31–33]. As a consequence, we characterize “impactful involvement” as involvement that empowers patients and public partners to contribute meaningfully [34].

All YPFC were invited to submit anonymous evaluations after each meeting, workshop, and training session. The evaluation tools used after YAG meeting #1–2 and YPRG Workshops #1–2 were evaluation questions modified to be specific to the project activities from the Public and Patient Engagement Evaluation Tool (PPEET) [35]. After YAG meeting #3, young people were invited to complete modified versions of the PPEET and the Patient Engagement In Research Scale (PEIRS) [35, 36]. They were also invited to share their reflections on project involvement through a *Jamboard* (Additional file 1, Section B).

For young people who participated in the Delphi study, they completed the evaluation surveys with questions modified to be specific to the Delphi study from the PPEET, PEIRS, and the Acceptability E-Scale [35–37]. They were also invited to attend an online post-Delphi debrief session to reflect on their experience and provide feedback with family caregivers who also completed the Delphi study.

For family caregivers, evaluation surveys comprised modified questions adapted to be specific to the project activities from various evaluation surveys, which included the PPEET, PEIRS, and the Acceptability E-Scale [35–37]. For the evaluation surveys administered after the Consensus Meeting and E&E, we also included a few questions on their perceived impact to the project. For each evaluation survey, a free text box was also available for family caregivers to elaborate on their responses or share openly about their experiences. We also conducted a debrief session at the end of the Delphi meeting, where we delved deeper on their experiences with the Delphi and to reflect on what helped them, what “worked”, or didn’t.

As the project progressed, the research team prospectively documented the impact of YPFC on the project materials, process, and deliverables.

### What worked and areas of improvement: key enablers of impactful involvement

We carefully reviewed the quantitative and qualitative feedback received from the evaluation surveys and debrief sessions. A general inductive approach was implemented [38], and feedback from YPFC were thematically combined to identify patterns and overarching themes. We factored in our reflections of YPFC involvement in this project, and recommendations published by Elsman et al. [14] and Manyara et al. [15]. Through this process, we identified overarching themes on what project practices worked well and on areas for improvement in facilitating the meaningful involvement of YPFC; these are supported by illustrative quotes from the evaluation feedback. The core research team then synthesized these themes into key enablers for meaningful, impactful involvement of YPFC in reporting guideline development.

We used the GRIPP2-Short Form (Guidance for Reporting Involvement of Patients and the Public) [39], included in Additional file 1, Section A to guide our reporting of the PPI in this project.

## Results

### Involvement experiences of young people

Overall, young people found their experience in the project to be valuable and worthwhile. All ratings from the evaluation surveys can be found in Additional file 1, Section D, 1–2; Section E, 1–2. After each YAG meeting, all advisors who attended the meeting responded to our invitation to give feedback, and they consistently indicated “strongly agree” or “agree” that each YAG meeting was a good use of their time and they were satisfied with the meeting, except for one youth advisor who rated their experience with the first meeting as “neutral”. Other elements of the evaluation, such as ease to attend, understandability, being able to express views, being provided sufficient support to join, and feeling confident that their input will be used by the research team, were consistently ranked as “strongly agree” or “agree”, with a few “neutral” ratings. This positive sentiment was also shared by young people who attended the YPRG workshops, with all attendees indicating that they strongly agree/agree that the workshop was a good use of their time and was an enjoyable experience. One attendee shared the following about their workshop experience:

“*I really enjoyed the workshop and thought that it was a great and useful conversation. I found the explanations very clear*,* and the different methods of discussion were great! (chat*,* Google Jamboard*,* talking).*” – YPRG Workshop Attendant.

**Fig. 2:**
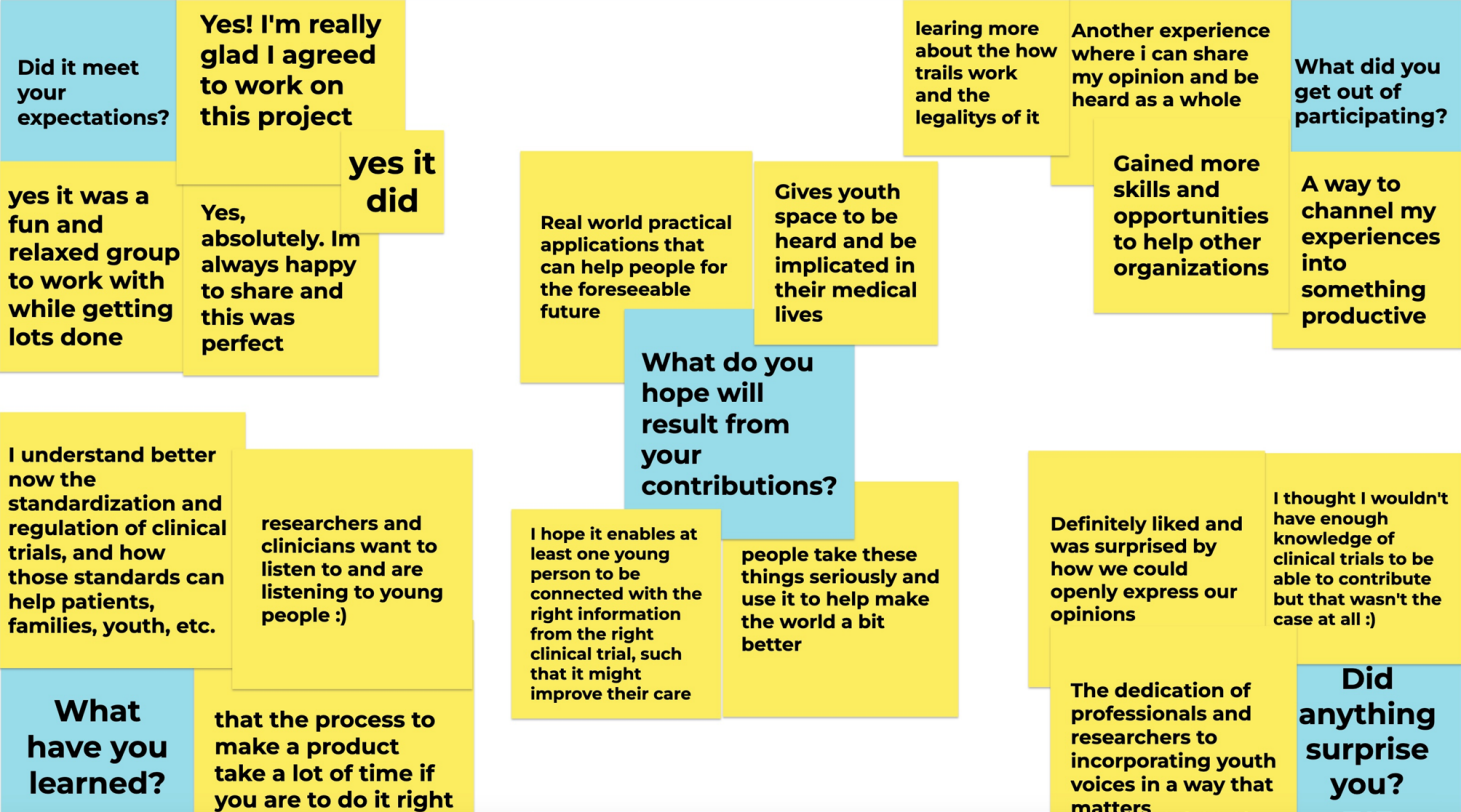
Youth Advisory Group’s reflection on their involvement

Reflections by the youth advisors at the final YAG meeting signified that being part of the advisory group and partaking in activities that they were interested in (i.e., YPRG workshop), was a positive experience (Fig. 2). Their reflections highlighted that being part of the project met their expectations, and they felt their experiences were able to help others and make a difference. They also learned more about RCTs and realized their voice and input was valued by researchers. Looking forward, they hoped their contributions to the project would help others in the future. Their overall sentiment is summarized below:

*“I’m always happy to share my experience*,* knowledge and opinion*,* especially when I am heard and listened [to]. This project let me do just that and I see my input being used in the project.”* – Youth Advisor, after final YAG meeting.

*“This was a fun way to help others and would love to continue help in this field.”* – Youth Advisor, after final YAG meeting.

The written evaluations provided some feedback related to improving project materials, and a few comments regarding background information provided during the YPRG workshops (Additional file 1, Section D, 1–2).

Young people who participated as Delphi panellists provided feedback on their experience with the Delphi, which is elaborated on more below and presented together with the feedback from family caregivers who completed the Delphi.

### Involvement experiences of family caregivers

Family caregiver advisors also had an overall positive experience being part of the FCAG. Similar to the youth advisors, all advisors indicated “strongly agree” or “agree” that each FCAG meeting was a good use of their time and was satisfactory. One family caregiver advisor shared their overall impressions:

*“It was such a fabulous learning experience*,* and I felt part of the wonderful team.”* – Family Caregiver Advisor, after final FCAG meeting.

Other elements of the evaluation, such as ease to attend, understandability, being able to express views, being provided sufficient supports to join, and feeling confident that their input will be used by the research team, were also consistently ranked as “strongly agree” or “agree”, with only one “neutral” rating regarding the ease to attend one of the meetings. The Consensus Meeting was also a highly regarded experience by the family caregiver advisors who attended. Qualitative and quantitative feedback received are detailed in Additional file 1, Section D, 3–6; Section E, 3–5.

The Delphi study involved family caregivers, both advisors (n = 5) and those who only participated in the Delphi study (n = 5), and young people (n = 4; ages 19–24 years). Most of the feedback received was positive, with the majority finding the training session helpful and a good use of their time:

*“From my point of view the training session was totally necessary. I would not have understood how to do the first survey without it. Before this exercise the word ‘Delphi’ was quite daunting for me. After the training*,* I had more confidence.” –* Family Caregiver Delphi Panellist, at the Post-Delphi Debrief/Focus Group.

Even after the Delphi study, most panellists rated the experience positively, except for one panellist disagreeing with the ease of completing the Delphi study, understandability of the content, and helpfulness of the training session. To better understand and dive deeper into their experiences with the Delphi study, a post-Delphi debrief session was conducted, which revealed areas that worked well and areas needing improvement, described below.

One project stage that was challenging for family caregivers was the E&E writing/review process. This process required working with other writing team members. Most of the other writing team members were not in the FCAG or involved in the YPFC involvement aspect of the project. Although family caregivers felt that they understood the objective of the E&E documents, learned new things, and improved their professional skills such as writing and research, the evaluations indicated that they felt the experience was more challenging than expected (Additional file 1, Section D, 6 and Section E, 6). Some felt they didn’t have enough experience to contribute meaningfully, while others shared that they felt that the process took significantly more time and dedication than anticipated to complete. They elaborated further on each of their ratings, which are available in Additional file 1, Section D, 6. Despite the challenges, they still felt that their input would make a difference to the guidelines. In response to whether being part of the E&E writing team was worth their time, one family caregiver shared:

*“I agree [that being part of the E&E writing team was worth my time]*,* even though I spent a lot more time than expected. It was worth my time because it aligned with some of my goals as a patient partner. I learned new skills and being part of this has a potential to contribute to better clinical trial reporting which in turn could improve the way paediatric clinical trials are done.”* – Family Caregiver Advisor, after the E&E writing process.

Feedback revealed what worked well and what didn’t, which informed enablers for involvement in reporting guideline development.

### Impact

The documented impact of YPFC on the project materials, process, and deliverables are detailed in Table 2.

**Table 2:** Impact of young people and family caregivers (YPFC) on the development of SPIRIT | CONSORT-Children & adolescents 2025

Project Stage	Impact* of the contributions of	
	Young people (ages 10–24 years)	Family caregivers
0. Planning	• Development of age-appropriate, understandable Young Person Reporting Guideline (YPRG) Workshop materials and approach; improved recruitment materials and workshop materials led to 42 young people (ages 10–21 years) from Canada, England, France, Scotland, and Spain joining and contributing their ideas at the YPRG workshops	• Development of understandable Delphi training session materials, Delphi survey instructions, survey format, and piloting survey; improved recruitment materials were used to identify additional family caregivers and young people (ages 19–24 years) to join the Delphi study, resulting in a total of 10 family caregivers and 4 young people (ages 19–24 years) signing up to be a Delphi panellist
1. Generation of candidate items	• Development of five new *Youth Generated* candidate reporting items and nine existing candidate reporting items voted on in Delphi Round 2; all were endorsed by youth during the YPRG workshops conducted simultaneously with Delphi Round 1	• Suggested potential new items for consideration during Delphi Study Round 1
2. Delphi study	• *Youth Endorsed* items and *Youth Generated* items clearly marked to flag items that reflect youth priorities, from Delphi Round 2 onwards • Two *Youth Generated* items voted in after the Delphi Study, and Two *Youth Generated* candidate items carried forward for discussion at the Consensus Meeting	• Improved wording clarity and optimized Delphi survey design through pilot testing Delphi Rounds 1 and 2 • Aggregated results from family caregivers and young people panellists displayed to all panellists to encourage reflection of YPFC priorities during voting • Shared feedback during the Post-Delphi survey, which resulted in lessons learned on what was effective and what could be improved for the Delphi study
3. Consensus Meeting	• Clearly flagged *Youth Generated* and *Youth Endorsed* candidate items during discussions; both remaining *Youth Generated* items discussed at the Consensus Meeting were voted in	• Attended the Consensus Meeting (*n* = 4) and contributed their lived experiences and actively contributed to discussions that informed voting of attendees and reflected their priorities
4. E&E writing	Excluded by design	• Contributed to writing and review of a total of 16 reporting items • Shared feedback on their experience, which resulted in lessons learned on how E&E writing/review processes can be optimized for patient and public partners in the future
5. Pilot testing	Excluded by design	Excluded by design
6. Finalization	• Acknowledgment of youth advisors who gave permission on the papers, recognizing their contributions to the project • Development of youth-appropriate materials based on the youth advisors’ input on appealing knowledge translation materials	• Critically reviewed the manuscript and became co-authors if criteria for based on the International Committee of Medical Journal Editors (ICMJE) were met • Development of dissemination materials appropriate for family caregivers • Dissemination of the guideline to their networks

*Impact is defined as the tangible changes, learnings, and outcomes, both positive and negative, on the guideline development process and to the final guidelines resulting from YPFC co-development

Throughout the project, contributions of YPFC had tangible impacts that were mostly positive. In brief, they were instrumental in the development of understandable project materials, such as workshop materials and Delphi surveys. Their input also positively impacted project processes such as identifying other YPFC to be involved. Importantly, YPFC impacted the relevance and credibility of the final deliverables. Their tangible impacts are evident through the generation and inclusion of four youth generated reporting items. This also applied to project publications, as family caregiver advisors were co-authors. Their contributions as co-authors resulted in the integration of their lived experiences in the Examples and Explanations of 16 reporting items in SPIRIT-C 2025 and CONSORT-C 2025, and in both statement papers.

Evaluation questions on perceived impact by family caregivers after the Consensus Meeting and E&E writing process indicated that they themselves felt they made a difference to the project. For both project stages, family caregivers indicated “strongly agree” or “agree” with regards to feeling like they were able to contribute, and that their input will make a difference in reporting guideline development (Additional file 1, Section E, 5, 7).

### Key enablers of impactful PPI

#### Overarching themes of what worked well and areas for improvement

Most of the qualitative feedback received in the free-text boxes in the evaluation forms came from family caregivers, as they were involved in more project stages compared to young people and had more opportunities to provide feedback. We also received qualitative feedback through debrief sessions and at meetings, which provided more depth and dimension to the evaluation survey results. What worked well included team composition, training and project materials, and openness of the research team. Areas for improvement included the need for training and examples, time, and organized check-in meetings. Each theme is supplemented by illustrative quotes from the feedback in eTables 2a and eTable 2b.

#### Six enablers of meaningful and impactful involvement

Our thematic synthesis yielded six key enablers to meaningfully involve YPFC in the development of reporting guidelines: (1) designated point person, (2) tailored training for all key project activities, (3) easy access to all project materials, (4) clear expectations on time commitment and compensation, (5) structured check-in meetings with team and others, and (6) demonstrated openness to feedback. Table 3 expands on these enablers with explanations on how to apply them in practice.

**Table 3:** Six key enablers for meaningful and impactful youth- and caregiver-partner-involvement in reporting guideline development

Enabler	Explanation	How to apply
1. Designated point **person**	Having a point person helped facilitate effective communication and was identified as one of the most important components in enabling effective participation throughout the project. For this project, the point person was part of the core project team and led the project and handled all logistics, such as scheduling meetings, organizing timely compensation, sending updates, reminders, and project materials. This point person was someone who partners could reliably reach out to and check in with, ask questions, and make suggestions to as well.	• Designate a point person who is knowledgeable on the project that young people and family caregivers can reach out to with questions and to check in • Involve the point person in coordinating the logistics of the project, including scheduling meetings, sending updates, reminders, project materials, and organizing timely compensation
2. Tailored **training** for all key project activities	Family caregivers, and young people who completed the Delphi study, identified onboarding and training sessions as vital. Scheduling dedicated sessions tailored to partners ensured everyone had a solid understanding of the project and its objectives, the necessary content, and their role. Prior to any project stage, such as joining the project, Delphi study, Consensus Meeting and E&E writing, we held an onboarding/training session to familiarize everyone with the why, what, and how of their involvement, with time for their questions. For the Delphi study, panellists shared how they would have appreciated more training, such as having a chance to practice the voting process during the training session. This sentiment was also shared for the E&E writing and review process. Inclusion of hands-on training for project stages that required partners to complete tasks by themselves over a long period of time, such as the Delphi study and the E&E writing process, was thought to be missing. Additionally, the availability of examples and exemplars that partners could refer to was something they identified as a potential useful resource to help in understanding concepts and furthering their understanding of the task at hand. For example, for the E&E writing process, having access to previously published E&E examples was identified as something that would have been helpful. Even for the Delphi study, family caregiver and young people panellists thought having more examples to work through together would have further enhanced their understanding.	• Customize training to each project activity, and include hands-on training opportunities for activities that may benefit from practical training (e.g., voting process for Delphi study, identifying examples and writing explanation texts for E&E) • Provide examples and exemplars that helps to put new concepts and theories into something tangible and understandable
3. Easy **access** to all project materials (e.g., slide deck, glossary)	Providing project materials, such as the meeting slide deck prior to meetings, was reportedly helpful. Having access to these materials allowed for better preparation and time to think ahead and formulate questions. We also emphasized that reviewing the material was optional and not required, so no one felt burdened to do so if they didn’t feel that it would be helpful to them. For the Delphi study, we provided a glossary that defined terms used throughout to all panellists, even those who were not family caregivers or young people. The glossary was available in two ways: (1) as a downloadable document, so users could refer to it or read it on their own time, and (2) for each question in the Delphi study, we provided a “glossary box” that defined the terms used in each reporting item on the same page. Family caregiver and young people Delphi panellists found both uses of the glossary helpful, as with the downloadable document they were able to read it on their own time, but when integrated directly in the Delphi survey, they didn’t have to spend time searching for the term(s).	• Provide project materials, such as the slide deck, in advance so those who are interested have ample time to review and formulate questions • Prepare a glossary that defines key terminology, have it available as a downloadable resource, but also consider integrating the glossary within project materials (e.g., glossary box within the Delphi study) for ease of use and access
4. Clear **expectations** on time commitment and compensation	A constant factor throughout the project was striking the balance of having sufficient time, to being too long and burdensome. Allocating sufficient time for preparation prior to the meeting, such as sending the slides with ample time for review, was identified as important. Particularly for the Delphi study, some felt that the time estimates to complete the survey was too short; additionally, some felt that they had to put in a lot of time to complete it. This was similar to the feedback received for the E&E writing process. Those who contributed as writers/reviewers felt that the process took longer than expected, and that the original timeline was too short. Therefore, providing time estimates that are realistic was seen as important for contributors to feel prepared prior to taking part in a project stage. We budgeted for compensation based on established criteria to recognize young people and family caregivers for their time and contributions, which were clearly communicated during meetings and in writing. Specifics are detailed further in eTable 1, Additional file 1.	• Provide realistic time estimates needed to complete various project stages • Budget for and clearly communicate planned compensation to recognize time and contributions of partners
5. Structured check-in **meetings** with team and others	Though we maintained open lines of communication throughout the project, for longer project stages, check in meetings that are organized ahead of time may be needed as a dedicated time to ask questions or touch base to see if they are on the right track. This was especially true for the E&E writing process, which spanned a period of two months. Having more structure in connecting with different team members, such as in the E&E writing process, to get everyone on the same page on tasks and deliverables is thought to have improved their experience.	• Build in time dedicated to touching base, where those who would like to can join to check in and connect with the research team • Schedule a time to introduce everyone to one another and lay out expectations and roles for one another for project stages that require partners to work with other collaborators
6. Demonstrated **openness to feedback**	Having a way to share their feedback and suggestions on what worked well and what didn’t, and having the project team be receptive to integrating changes was appreciated by partners. Being able to provide feedback through various means, such as through evaluation surveys and during meetings was reportedly effective. Maintaining open lines of communication to allow for mutual learning and improvement was a priority for the research team, but evidently also something that partners valued.	• Be open to receiving feedback and adjusting to changes throughout the process • Provide multiple ways for young people and family caregivers to provide feedback (e.g., anonymous evaluation surveys, including multiple choice and free-text box; interactive whiteboards; focus groups; e-mail feedback at a time that works for them)

## Discussion

Sharing and reporting methods on how to effectively involve YPFC in reporting guideline development can serve as helpful resources for future researchers in developing plans and methods on how to effectively involve these key partners. To date, there are few instances of YPFC involvement in research that are well-reported [40]. We aimed to thoroughly evaluate, describe, and report the involvement, experience and impact of YPFC in the development of paediatric research reporting guidelines to identify enablers of meaningful and impactful involvement.

### Involvement experience, impact, and enablers

#### Involvement experience

We successfully involved 50 young people and 10 family caregivers in the development of the SPIRIT-C 2025 and CONSORT-C 2025 by implementing Elsman et al. [14] and Manyara et al.’s [15] recommendations. Key facilitators instrumental in developing an effective involvement and evaluation strategy included: (a) lessons learned from our previous experiences working with young people and families [23, 24], (b) embedding a person with lived experience and expertise in youth and family engagement in the core project team, (c) the expertise of the International Youth Involvement Steering Committee, and (d) the implementation of published recommendations specific to working with PPI in reporting guideline development. Many of the identified overarching themes of what worked well in supporting YFPC involvement, such as team composition, training, access to project materials (i.e., glossary), and being able to share feedback openly, were made possible through the implementation of the published recommendations and lessons learned.

However, despite following the “blueprint” recommendations (eTable 1), the involvement experience and evaluation feedback revealed areas that needed further improvement. For one, although the availability of training was thought to work well, it was also an area that could benefit from further improvement with more customized training based on the specific project activity. In addition, while we were cognizant of the importance of setting a timeframe needed for the Delphi surveys as based on Elsman et al.’s recommendations, the experiences and feedback received made it evident that realistic time estimates need to be provided for all project stages. Notably, based on the challenges reported by the family caregiver advisors on the E&E writing process, it is evident that aside from realistic time estimates, proper consideration of the skills and responsibilities necessary for certain project stages is needed. Depending on the experience and expertise of each patient and public partner, project stages that require technical skills, such as academic writing, may pose a barrier to meaningful involvement. The integration of tailored, hands-on training reflective of each project stage is insightful for both researchers and patient and public partners to determine the need for more support, or whether full participation in the activity is feasible and meaningful.

Relatedly, this further reinforces the core research team’s decision against inviting young people to the Consensus Meeting. This decision was made as the meeting took place during weekday school hours, and attending a highly technical, five-hour meeting may not have been the most conducive use of young people’s time. Furthermore, most of the youth workshop leaders (JP, BNE, SG) were present at the Consensus Meeting.

#### Impact

In the development of SPIRIT-C 2025 and CONSORT-C 2025, YPFC positively impacted project processes, outcomes, and deliverables, as evident in their impact detailed in Table 2. Partnering with YPFC resulted in the emphasis on family and patient-centred practices in RCTs, including consent/assent processes, pain reduction, and reducing potential harms, which are now reflected in SPIRIT-C 2025 and CONSORT-C 2025 [21, 22]. Attention to these areas and the integration of YPFC priorities has downstream effects and should improve reporting of paediatric RCT protocols and reports; additionally, it could positively impact continued participation and experiences of children, young people, and families in trials.

Impact of PPI, and how to assess this, is an area of interest [31, 41]. The practicalities and feasibility of involving young people and families in reporting guideline development is still unknown. Limited reporting of authentic involvement of young people throughout the research process is available [18, 40], and while some studies report positive experiences and impact of young people’s involvement in research, most do not use formal evaluations to assess this [18]. A main reason for this is that while there is growing interest in PPI in research, a standard process on how to assess impact of PPI on research does not exist [30, 31, 41]. A recently published narrative review synthesized the impact of young people on research to the following: influence on research, outputs resulting from their involvement, shifts in the attitudes of researchers, feeling of achievement in young people, and development of deeper relationships [42]. To fully appreciate the contributions of YPFC to the development of research reporting guidelines, evaluating the impact of their contributions may provide insight on how they contributed, and how they may have affected the credibility, usability, and understandability of the final guidelines. Although there are published accounts of how young people have impacted research [18, 42], there is no accepted definition for “involvement impact”.

The impact of PPI is context dependent, leading some to argue the generic value of this measurement [43]. Others argue that measuring impact may lead to performativity, emphasizing the need for a balanced evaluation of both positive and negative impacts of PPI [30]. For this project, evaluating the impact of YPFC in the development of paediatric reporting guidelines in the context of their experiences was critical in understanding key enablers for their impactful involvement in reporting guideline development, and the implications of their involvement on (a) paediatric research and (b) best practices in working with YPFC.

#### Enablers: Implications for paediatric research

Evidently, impactful involvement of PPI in paediatric research has many benefits, including increasing the relevance and utility of the research findings, and fostering buy-in from key partners in the research process, such as increased retention of participants [10, 16–18]. Their involvement provides valuable perspectives, and everyone’s unique expertise, education, and experiences can immensely enrich the project outcomes and deliverables, thus reducing research waste [42, 44]. The SPIRIT-C 2025 and CONSORT-C 2025 guidelines have benefitted from the involvement of YPFC, with improved relevance, utility, and credibility, as demonstrated through their impact and feedback. The application of the identified key enablers in future paediatric research can facilitate YPFC to be meaningfully involved, so that their perspectives are heard and valued.

Meaningfully involving YPFC gives them the opportunity to not only positively impact project processes, materials, and deliverables, but also impact the people involved. For one, involving young people in research has been reported to be an empowering and enjoyable opportunity that provides them with experiences to learn, gain skills, knowledge, and network [18, 42, 45]. Young people are also able to develop as individuals, and involvement in research reportedly can build up their confidence and self-esteem [18, 42, 45]. Reflections from the young people involved in this present project demonstrated that these practical values and benefits were experienced and appreciated (Fig. 2). Integration of PPI in research can facilitate research teams to gain new skills and knowledge, broaden networks and community, and strengthen their relationship with patients and families [16, 18, 42].

#### Enablers: Implications for best PPI practice

Meaningful and impactful involvement of YPFC is gradually being recognized as part of responsible research practice [14, 15, 23, 46, 47]. Authentic involvement of young people in decision making processes that affect their lives is part of their rights, as per the UNCRC [11, 48]. Therefore, identifying enablers that foster impactful involvement of YPFC is needed, and can be understood through their involvement experiences, evaluation feedback, and the impact of their contributions.

The six key enablers – designated point person, tailored training, access to project materials, clear expectations on time commitment and compensation, structured check-in sessions, and demonstrated openness to feedback – were synthesized from the overarching themes of what worked well, and areas that need improvement. These enablers are vital considerations for future projects interested in working with YPFC in the development of reporting guidelines. Development of research reporting guidance involves the need to understand complex and abstract concepts, which necessitates careful planning and consideration particularly when working with young people and families. This is demonstrated in various other methodological paediatric research initiatives involving YPFC, including the development of core outcome sets, trial design, and trial results communication tools [14, 15, 23, 46, 47]. The six key enablers encourage thoughtful planning of YPFC’s involvement, which is integral to key partners being involved purposefully as partners, and ultimately to facilitate them in contributing in ways that are meaningful and impactful. Beyond these six key enablers, proper training of research teams on how to meaningfully involve patient and public partners is needed. The needs for training of researchers in the proper conduct of PPI have long been recognized [49–51]. Depending on the researcher’s background and experience, tailored training for them should also be planned.

### Limitations

There are some limitations with this study. We recognize that there is heterogeneity in the way we refer to youth and young people. This is due to the existing variability in the ways advisory groups are referred to globally and in age groups covered under different terms. Additionally, a well-cited challenge in research that involve patient and public partners is representation. We recognize one of the limitations of this work is that the YPFC involved in this project were selected based on their background and expertise, which are likely not representative of the general population. However, due to the nature and content of the project, we elected to include partners with relevant background and experiences, so they can meaningfully contribute to the project. Relatedly, as we mostly involved established YPAG groups, the perspectives of young people involved in the project may not be representative of those who are not engaged with YPAGs or other similar research groups. Due to the complexity of the concepts in this present project, we elected to involve YPAG groups with sufficient experience in clinical trial research. Nevertheless, more efforts need to be made to make research involvement more diverse and accessible. In addition, despite our efforts to involve family caregivers outside of Canada in the Delphi study, only family caregivers from Canada signed up to partake in the Delphi study. Lastly, though most of the Family Caregiver Advisors were involved as co-authors in this paper, we did not involve Youth Advisors as co-authors in this work, considering the complexity of the paper and original stated expected workload to our advisors, which did not include the lengthy writing and review process. Future work is needed in this area to explore how young people can be meaningfully involved in the writing process, with consideration of their expertise and interest, which will be informative for both young people and researchers.

## Conclusion

Through integration of recommendations from multiple sources, we designed a tailored approach to involve YPFC in the development of paediatric RCT reporting guidelines. Their involvement was mutually beneficial, was a positive experience that was valued by all involved and resulted in tangible impacts on the project. The identified enablers for meaningful involvement can serve as a resource for future projects aiming to involve YPFC in an impactful way in methodological paediatric research such as reporting guideline development.

## Electronic supplementary material

Below is the link to the electronic supplementary material.

Supplementary Material 1

## Data Availability

All data supporting the findings are available within the paper and Additional file 1

## Abbreviations

CONSORT: Consolidated Standards of Reporting Trials
CONSORT-C: CONSORT-Children and Adolescents
COSMIN: COnsensus-based Standards for the selection of health Measurement INstruments
E&E: Explanation and Elaboration
FCAG: Family Caregiver Advisory Group
GRIPP-2 SF: Guidance for Reporting Involvement of Patients and the Public-2 Short Form
LMIC: Low-middle income countries
OMI: outcome measurement instrument
PEIRS: Patient Engagement In Research Scale
PPEET: Public and Patient Engagement Evaluation Tool
PPI: Patient and public involvement
PRISMA: Preferred Reporting Items for Systematic Reviews and Meta-Analyses
RCT: Randomised controlled trial
SPIRIT: Standard Protocol Items for Interventional Trials
SPIRIT-C: SPIRIT-Children and Adolescents
UNCRC: United Nations Convention on the Rights of the Child
WHO: World Health Organization
YAG: Youth Advisory Group
YPFC: Young People and Family Caregivers
YPRG: Young Person Reporting Guideline

## References

[1] Clyburne-Sherin AV, Thurairajah P, Kapadia MZ, Sampson M, Chan WW, Offringa M. Recommendations and evidence for reporting items in pediatric clinical trial protocols and reports: two systematic reviews. Trials. 2015;16:417. 26385379 10.1186/s13063-015-0954-0 PMC4574457

[2] Series from the Lancet journals: Research: increasing value, reducing waste: The Lancet. 2014 [Available from: https://www.thelancet.com/series/research

[3] Hopewell S, Boutron I, Chan AW, Collins GS, de Beyer JA, Hrobjartsson A, et al. An update to SPIRIT and CONSORT reporting guidelines to enhance transparency in randomized trials. Nat Med. 2022;28(9):1740–3. 36109642 10.1038/s41591-022-01989-8

[4] Li Q, Zhou Q, Florez ID, Mathew JL, Amer YS, Estill J et al. Reporting standards for child health research were few and poorly implemented. J Clin Epidemiol. 2023. 10.1016/j.jclinepi.2023.03.017 36965601

[5] EQUATOR Network. Enhancing the QUAlity and Transparency Of health Research [Available from: http://www.equator-network.org/reporting-guidelines/

[6] Bhaloo Z, Adams D, Liu Y, Hansraj N, Hartling L, Terwee CB, et al. Primary outcomes reporting in trials (PORTal): a systematic review of inadequate reporting in pediatric randomized controlled trials. J Clin Epidemiol. 2017;81:33–41. 27667370 10.1016/j.jclinepi.2016.09.003

[7] Gates A, Hartling L, Vandermeer B, Caldwell P, Contopoulos-Ioannidis DG, Curtis S, et al. The conduct and reporting of child health research: an analysis of randomized controlled trials published in 2012 and evaluation of change over 5 years. J Pediatr. 2018;193:237–44. e37. 29169611 10.1016/j.jpeds.2017.09.014

[8] Saint-Raymond A, Hill S, Martines J, Bahl R, Fontaine O, Bero L. Consort 2010. Lancet. 2010;376(9737):229–30. 20656114 10.1016/S0140-6736(10)61134-8

[9] Baba A, Offringa M. Transparent Reporting-SPIRIT-C/CONSORT-C pediatric updates. JAMA Pediatr. 2023. 10.1001/jamapediatrics.2023.5740 38147343

[10] Sammy A, Baba A, Klassen TP, Moher D, Offringa M. A decade of efforts to add value to child health research practices. J Pediatr. 2023;265:113840. 38000771 10.1016/j.jpeds.2023.113840

[11] United Nations Convention on the Rights of the Child. General Comment No. 12: The right of the child to be heard. 2009.

[12] Butcher NJ, Monsour A, Mew EJ, Chan A-W, Moher D, Mayo-Wilson E, et al. Guidelines for reporting outcomes in trial protocols: the SPIRIT-Outcomes 2022 extension. JAMA. 2022;328(23):2345–56. 36512367 10.1001/jama.2022.21243

[13] Butcher NJ, Monsour A, Mew EJ, Chan A-W, Moher D, Mayo-Wilson E, et al. Guidelines for reporting outcomes in trial reports: the CONSORT-Outcomes 2022 extension. JAMA. 2022;328(22):2252–64. 36511921 10.1001/jama.2022.21022

[14] Elsman EBM, Smith M, Hofstetter C, Gavin F, Jobson E, Markham S, et al. A blueprint for patient and public involvement in the development of a reporting guideline for systematic reviews of outcome measurement instruments: PRISMA-COSMIN for omis 2024. Res Involv Engagem. 2024;10(1):33. 38515153 10.1186/s40900-024-00563-5 PMC10956212

[15] Manyara AM, Stewart D, Markham S, Worrall A, Harris R, Davies P et al. Patient and Public Involvement in methodology research: Process, experiences, reflections, and recommendations from the SPIRIT | CONSORT-Surrogate project. Under review. 10.1186/s40900-025-00807-y PMC12720433 41345899

[16] Vanderhout SM, Bhalla M, Van A, Fergusson DA, Potter BK, Karoly A, et al. The impact of patient and family engagement in child health research: A scoping review. J Pediatr. 2023;253:115–28. 36179891 10.1016/j.jpeds.2022.09.030

[17] Baba A, Aregbesola A, Caldwell PHY, Elliott SA, Elsman EBM, Fernandes RM et al. Developments in the design, conduct, and reporting of child health trials. Pediatrics. 2024;154(1). 10.1542/peds.2024-065799 38832441

[18] Warraitch A, Wacker C, Biju S, Lee M, Bruce D, Curran P, et al. Positive impacts of adolescent involvement in health research: an umbrella review. J Adolesc Health. 2024;75(2):218–30. 38597838 10.1016/j.jadohealth.2024.02.029

[19] Chan A-W, Boutron I, Hopewell S, Moher D, Schulz KF, Collins GS, et al. SPIRIT 2025 statement: updated guideline for protocols of randomised trials. BMJ. 2025;389:e081477. 40294953 10.1136/bmj-2024-081477 PMC12035670

[20] Hopewell S, Chan A-W, Collins GS, Hróbjartsson A, Moher D, Schulz KF, et al. CONSORT 2025 statement: updated guideline for reporting randomised trials. BMJ. 2025;389:e081123. 40228833 10.1136/bmj-2024-081123 PMC11995449

[21] Baba A, Smith M, Potter B, Chan A-W, Moher D, Toulany A et al. Enhancing the reporting and impact of paediatric randomised trials: CONSORT-Children and adolescents (CONSORT-C) 2025 extension. Under review.

[22] Baba A, Smith M, Potter B, Chan A-W, Moher D, Toulany A et al. Enhancing the reporting and usefulness of paediatric randomised trial protocols: SPIRIT-Children and adolescents (SPIRIT-C) 2025 extension. Under review.

[23] Baba A, Richards DP, Smith M, Pallone N, Vanderhout S, Prebeg M, et al. Youth and family involvement in the development of a plain Language trial results communication tool: communikids. Res Involv Engagem. 2023;9(1):88. 37777802 10.1186/s40900-023-00499-2 PMC10544151

[24] Prebeg M, Patton M, Desai R, Smith M, Krause K, Butcher N, et al. From participants to partners: reconceptualising authentic patient engagement roles in youth mental health research. Lancet Psychiatry. 2023;10(2):139–45. 36502816 10.1016/S2215-0366(22)00377-7

[25] World Health Organization. Adolescent health in the South-East Asia Region Geneva: World Health Organization; [Available from: https://www.who.int/southeastasia/health-topics/adolescent-health

[26] World Health Organization. Adolescent health [Available from: https://www.who.int/health-topics/adolescent-health#tab=tab_1

[27] United Nations. Convention on the Rights of the Child 1989 [Available from: https://www.ohchr.org/en/instruments-mechanisms/instruments/convention-rights-child

[28] World Health Organization. Newborn health [Available from: https://www.who.int/westernpacific/health-topics/newborn-health#:~:text=A%20newborn%20infant%2C%20or%20neonate,to%20health%20care%20is%20low

[29] Brett J, Staniszewska S, Mockford C, Herron-Marx S, Hughes J, Tysall C, et al. Mapping the impact of patient and public involvement on health and social care research: a systematic review. Health Expect. 2014;17(5):637–50. 22809132 10.1111/j.1369-7625.2012.00795.x PMC5060910

[30] Russell J, Fudge N, Greenhalgh T. The impact of public involvement in health research: what are we measuring? Why are we measuring it? Should we stop measuring it? Res Involv Engagem. 2020;6:63. 33133636 10.1186/s40900-020-00239-w PMC7592364

[31] Crocker JC, Boylan AM, Bostock J, Locock L. Is it worth it? Patient and public views on the impact of their involvement in health research and its assessment: a UK-based qualitative interview study. Health Expect. 2017;20(3):519–28. 27338242 10.1111/hex.12479 PMC5433537

[32] National Institute for Health and Care Research. Impact and evaluation Learning for Involvement2024 [Available from: https://www.learningforinvolvement.org.uk/topic/evaluating-involvement/#:~:text=At%20NIHR%2C%20we%20would%20define,%2C%20carers%20and%20the%20public).

[33] PiiAF Study Group. PiiAF Glossary [Available from: https://piiaf.org.uk/glossary.php

[34] Dews SA, Bassi A, Buckland S, Clements L, Daley R, Davies A, et al. Characterising meaningful patient and public involvement in the pharmaceutical industry research setting: a retrospective quality assessment. BMJ Open. 2023;13(8):e071339. 37612107 10.1136/bmjopen-2022-071339 PMC10450071

[35] Abelson J, Li K, Wilson G, Shields K, Schneider C, Boesveld S. Supporting quality public and patient engagement in health system organizations: development and usability testing of the public and patient engagement evaluation tool. Health Expect. 2016;19(4):817–27. 26113295 10.1111/hex.12378 PMC5152717

[36] Hamilton CB, Hoens AM, McQuitty S, McKinnon AM, English K, Backman CL, et al. Development and pre-testing of the patient engagement in research scale (PEIRS) to assess the quality of engagement from a patient perspective. PLoS ONE. 2018;13(11):e0206588. 30383823 10.1371/journal.pone.0206588 PMC6211727

[37] Tariman JD, Berry DL, Halpenny B, Wolpin S, Schepp K. Validation and testing of the acceptability E-scale for web-based patient-reported outcomes in cancer care. Appl Nurs Res. 2011;24(1):53–8. 20974066 10.1016/j.apnr.2009.04.003 PMC3030937

[38] Thomas DR. A general inductive approach for analyzing qualitative evaluation data. Am J Evaluation. 2006;27(2):237–46.

[39] Staniszewska S, Brett J, Simera I, Seers K, Mockford C, Goodlad S, et al. GRIPP2 reporting checklists: tools to improve reporting of patient and public involvement in research. BMJ. 2017;358:j3453. 28768629 10.1136/bmj.j3453 PMC5539518

[40] Preston J, Biglino G, Harbottle V, Dalrymple E, Stalford H, Beresford MW. Reporting involvement activities with children and young people in paediatric research: a framework analysis. Res Involv Engagem. 2023;9(1):61. 37525218 10.1186/s40900-023-00477-8 PMC10388467

[41] PiiAF Study Group. The Public Involvement Impact Assessment Framework: Executive Summary. 2014.

[42] Thomas C, Cockcroft E, Jenkins G, Liabo K. Working with children and young people in research: supportive practices and pathways to impact. J Child Health Care. 2023:13674935231171451. 10.1177/13674935231171451 PMC11874604 37186542

[43] Staley K. Is it worth doing?’ measuring the impact of patient and public involvement in research. Res Involv Engagem. 2015;1:6. 29062495 10.1186/s40900-015-0008-5 PMC5598089

[44] Preston J, Lappin E, Ainsworth J, Wood CL, Dimitri P. Involving children and young people as active partners in paediatric health research. Paediatrics Child Health. 2024;34(1):11–6.

[45] Nguyen L, van Oort B, Davis H, van der Meulen E, Dawe-McCord C, Franklin A, et al. Exploring the how in research partnerships with young partners by experience: lessons learned in six projects from canada, the netherlands, and the united Kingdom. Res Involv Engagem. 2022;8(1):62. 36397131 10.1186/s40900-022-00400-7 PMC9672637

[46] Monga S, Monsour A, Stallwood E, Desai R, Cleverley K, Courtney D et al. Core outcome set development for adolescent major depressive disorder clinical trials: A registered report. JAACAP. 2020. 10.1016/j.jaac.2020.07.905 33126995

[47] Monga S, Desai R, Anthony SJ, Arnold PD, Bagnell A, Birmaher B, et al. Study preregistration: measuring what matters: development and dissemination of a core outcome set for pediatric anxiety disorders clinical trials. J Am Acad Child Adolesc Psychiatry. 2023;62(6):696–8. 37244653 10.1016/j.jaac.2023.01.016

[48] Checkoway B. What is youth participation? Child Youth Serv Rev. 2011;33(2):340–5.

[49] Dudley L, Gamble C, Allam A, Bell P, Buck D, Goodare H, et al. A little more conversation please? Qualitative study of researchers’ and patients’ interview accounts of training for patient and public involvement in clinical trials. Trials. 2015;16:190. 25928689 10.1186/s13063-015-0667-4 PMC4410574

[50] de Wit M, Beurskens A, Piskur B, Stoffers E, Moser A. Preparing researchers for patient and public involvement in scientific research: development of a hands-on learning approach through action research. Health Expect. 2018;21(4):752–63. 29418053 10.1111/hex.12671 PMC6117481

[51] Yu R, Hanley B, Denegri S, Ahmed J, McNally NJ. Evaluation of a patient and public involvement training programme for researchers at a large biomedical research centre in the UK. BMJ Open. 2021;11(8):e047995. 34385250 10.1136/bmjopen-2020-047995 PMC8362711

