# Supplementary material for: Experiences, impact, and enablers of involving young people and family caregivers in developing reporting guidelines for paediatric randomised trials: a case study
Source: Res Involv Engagem. 2025 Jul 1;11:71. doi: 10.1186/s40900-025-00751-x (PMC12211127; doi:10.1186/s40900-025-00751-x)
Supplement: Supplementary file 1 — Supplementary Material 1 [file 40900_2025_751_MOESM1_ESM.docx]

**Experiences, impact, and enablers of involving young people and family caregivers in developing reporting guidelines for paediatric randomised trials: a case study**

**Revised version, June 2025**

**Additional File 1**

Table of Contents

[A. GRIPP2-SF: Guidance for Reporting Involvement of Patients and the Public – Short Form 3](#_Toc199339587)

[B. Involvement of Young People (ages 10-24 years) 3](#_Toc199339588)

[1. Youth Advisory Group 3](#_Toc199339589)

[Meeting #1 – Onboarding session 4](#_Toc199339590)

[Meeting #2 – YPRG workshop materials 4](#_Toc199339591)

[Meeting #3 – Ideas on knowledge translation, personal reflections 5](#_Toc199339592)

[2. Young Persons Reporting Guideline (YPRG) Workshops 5](#_Toc199339593)

[Workshop planning 6](#_Toc199339594)

[Identification of workshop attendees 6](#_Toc199339595)

[Workshop #1: Project introduction and focus on SPIRIT-C 2025 7](#_Toc199339596)

[Workshop #2: Continuing the discussion and focus on CONSORT-C 2025 7](#_Toc199339597)

[Post-Workshop 8](#_Toc199339598)

[3. Delphi study 8](#_Toc199339599)

[C. Involvement of Family Caregivers 8](#_Toc199339600)

[1. Formation of the Family Caregiver Advisory Group (FCAG) 8](#_Toc199339601)

[Meeting #1 – Onboarding session 8](#_Toc199339602)

[Meeting #2 – Advice on Delphi study materials 9](#_Toc199339603)

[Meeting #3 – Consensus meeting and E&E Prep 9](#_Toc199339604)

[Meeting #4 – Knowledge translation and reflections 9](#_Toc199339605)

[2. Delphi Study 9](#_Toc199339606)

[Delphi Study Training 10](#_Toc199339607)

[3. Consensus Meeting 10](#_Toc199339608)

[Pre-Consensus Preparatory Meeting (and E&E writing process training) 11](#_Toc199339609)

[4. Explanation and Elaboration (E&E) Writing Process 11](#_Toc199339610)

[D. Qualitative Feedback from Evaluation Surveys, by Key Partner Group 12](#_Toc199339611)

[1. Feedback from Youth Advisory Group 12](#_Toc199339612)

[2. Feedback from Young Person Reporting Guideline Workshops 13](#_Toc199339613)

[3. Feedback from Family Caregiver Advisory Group 14](#_Toc199339614)

[4. Feedback from Delphi Panellists 16](#_Toc199339615)

[5. Feedback after Consensus Meeting 23](#_Toc199339616)

[6. Feedback after E&E writing process 24](#_Toc199339617)

[E. Quantitative Feedback from Evaluation Surveys: Heatmaps 28](#_Toc199339618)

[1. Feedback from Youth Advisory Group 28](#_Toc199339619)

[2. Feedback from Young Person Reporting Guideline Workshops 30](#_Toc199339620)

[3. Feedback from Family Caregiver Advisory Group 31](#_Toc199339621)

[4. Feedback from Delphi Panellists 33](#_Toc199339622)

[5. Feedback from Consensus Meeting Attendees (Family Advisors) 34](#_Toc199339623)

[6. Feedback after Explanation and Elaboration (E&E) Writing Process 35](#_Toc199339624)

[F. eTables and eFigure 36](#_Toc199339625)

[eTable 1. Twenty-one applications of 17 “Blueprint” recommendations (3) in the development of SPIRIT | CONSORT-Children & Adolescents 2025 36](#_Toc199339626)

[eTable 2a. What worked well, with illustrative quotes from feedback received 41](#_Toc199339627)

[eTable 2b. Areas for improvement with illustrative quotes from feedback received 43](#_Toc199339628)

[eFigure 1: Blueprint recommendations for involving patient and public partners in reporting guideline development (3) 44](#_Toc199339629)

[References 45](#_Toc199339630)

# **A. GRIPP2-SF: Guidance for Reporting Involvement of Patients and the Public – Short Form**

| **Section: topic** | **Item** | **Reported on page** |
| --- | --- | --- |
| 1: Aim | Report the aim of PPI in the study | Introduction |
| 2: Methods | Provide a clear description of the methods used for PPI in the study | Methods, Table 1, Figure 1, Additional file 1 |
| 3: Study results | Outcomes – Report the results of PPI in the study, including both the positive and negative outcomes | Results, Table 2-3, Figure 2, Discussion, & Additional file 1 |
| 4: Discussion and conclusions | Outcomes – Comment on the extent to which PPI influenced the study overall. Describe positive and negative effects. | Results, Discussion, Additional file 1 |
| 5: Reflections/critical perspective | Comment critically on the study, reflecting on the things that went well and those that did not, so others can learn from this experience. | Discussion |

# **B. Involvement of Young People (ages 10-24 years)**

The SPIRIT | CONSORT-C core project team included a patient engagement expert (MS), who was actively involved throughout the entire project. As a patient partner and citizen leader with extensive experience in Patient and Public Involvement (PPI), which includes engaging young people and families in methodological research, MS co-led the development of the young people and family caregiver involvement strategy in the SPIRIT | CONSORT-C project. MS also has lived experience of being a participant of paediatric trials due to a rare disease diagnosis in childhood. We formed an International Youth Involvement Steering Committee with members of the SPIRIT | CONSORT-C core project team (MS, AB, MO), and the European Young Person Advisory Group Network (eYPAGnet; JP, BNE, SG, PD). The eYPAGnet was founded by youth facilitator experts who lead work in PPI with children, young people, and families across different European countries. The network is renowned for setting up and working with children and young people through the forum of Young People’s Advisory Groups (YPAGs) and is founded by the following groups: GenerationR (England), Kids Barcelona (Spain), Kids France (Lyon), and ScotCRN (Scotland). The International Youth Involvement Steering Committee met several times to discuss and plan how to meaningfully involve youth in the SPIRIT | CONSORT-C project, with consideration of all steering committee members’ experience and expertise in working with their local YPAGs and in different research projects involving youth. In parallel, members of the core project team (AB, MO, MS) held a consultation with the KidsCan YPAG based in Vancouver, Canada, on their thoughts on 1) how young people can help make the SPIRIT | CONSORT-C project successful, 2) what would make young people want to be involved in the project, and 3) what would help young people become involved (e.g., training opportunities, compensation).

## **1. Youth Advisory Group**

The six members of the YAG (ages 13-19 years; average: 16.6 years) who were interested in being involved throughout the project and had a) lived experience of being a paediatric trial participant, b) who read research trial results to inform decision making for their own healthcare, and/or c) those who have worked with researchers as a patient/public partner. Youth advisors resided in various cities in Alberta (n = 2), British Columbia (n = 2), Ontario (n = 1), and Quebec (n = 1). The YAG represented the voices of young people, and their role was to advise on the design and content of the Young Person Reporting Guideline (YPRG) workshops, and in developing a knowledge translation strategy of the project deliverables to other young people. Those who were involved in the YAG also had the option of contributing to other project stages that they advised on, such as the YPRG workshops, detailed below. The YAG met virtually three times (1 hour each) from October 2023 – May 2024.

To form the YAG, recruitment materials were circulated to three groups in Canada: KidsCan YPAG, the INFORM RARE Youth Advisory Group, and the CommuniKIDS Youth Advisors. Nine young individuals (ages 13-20 years) expressed their interest through an online survey, and members of the core project team (AB, MS, MO) reviewed all responses and invited seven respondents to join the first YAG meeting based on respondents’ lived experience with paediatric trials, working with research teams in an advisory capacity, and their age, to create a YAG with diversified experiences and perspectives. A YAG with six young people was formed. In between meetings, the project team communicated with the advisors through e-mail regarding compensation, scheduling, and answering any questions about the project and their involvement. Details of each meeting are outlined below.

### **Meeting #1 – Onboarding session**

The first YAG meeting was an onboarding session where YAG members were introduced to the project and the role of the YAG, including expected timeline/hourly commitment, compensation, and the different ways they could get involved in the project should they not want to commit as an advisor. The meeting was facilitated by members of the core project team (MS, AB, MO). At the end of the hour, a brief discussion took place with all attendees on how much information on randomised controlled trials (RCTs) they thought youth their age would need to know in order to think about what is important to report about a RCT. Youth shared with us that having a rough template on how a RCT works, and what to expect, with the steps of a trial would be helpful. After the meeting, all attendees were asked to complete an evaluation form to provide feedback on the meeting and the recruitment materials that were used to recruit YAG members, as these materials were going to be adapted for recruitment of other young people to the YPRG Workshop. They were also asked to fill out a post-onboarding interest form to confirm their interest in being an advisor on the YAG, and to indicate whether they also want to join the YPRG Workshops. Of the six who attended the onboarding session, all of them committed to being advisors on the YAG, and five advisors expressed interest in also joining the YPRG workshops. Feedback received on the evaluation form was overall positive, and the YPRG provided constructive criticism on ways to improve the recruitment material to improve readability and provide necessary information.

### **Meeting #2 – YPRG workshop materials**

The second YAG meeting started off with advisors anonymously sharing why they joined the project on Jamboard. Many of their reasons for contributing were related to wanting to help due to their own experiences with their health, RCTs, and the healthcare system, and with the desire of learning more about RCTs and bettering the future. If they wished, advisors were given the opportunity to share with the group about their health conditions, their experiences with RCTs, and/or working collaboratively with researchers.

Based on feedback from the first YAG meeting, and discussions with the International Youth Involvement Steering Committee (detailed below, under “Workshop planning”), we prepared a draft slide deck containing potential material to be used in the YPRG workshops. We created a draft storyboard about two paediatric migraine trialists who were 1) planning a trial, and 2) had conducted a trial and were reporting its results. We then presented two personas from the perspective of youth who were 1) looking for a trial to join for their health condition and 2) reading a trial report about a new medication for their own health condition. Advisors shared their thoughts on the presentation and formatting of the two RCT stories and the personas, the level of detail provided, and suggested changes; their impact on the materials are detailed in Table 2. At the end of the meeting, we showed them a “What we heard, what we did” summary of their comments on the recruitment material, and how their comments shaped the recruitment material developed for the recruitment of attendees for the YPRG workshops.

### **Meeting #3 – Ideas on knowledge translation, personal reflections**

The objectives of the third YAG meeting were to share project updates and the impact of youth contributions to workshop planning, hear advisors’ thoughts on the best way to translate the knowledge from the SPIRIT | CONSORT-C project to young people and families, and to reflect on their involvement in the project.

Advisors favored a combination of an infographic and video as the most effective way to let young people and families know about these guidelines, and how they could use them. Their reasoning for these preferences included the ability for infographics to contain specifics that could be read at an individual’s own pace, while videos with segments from youth advisors could add a personal touch and credibility to the project. Advisors emphasized that the infographic needs to be visually appealing for youth, and not too “science-y”. To increase uptake of both, they suggested the addition of a QR code that would lead readers of the infographic to the video, and a link to the infographic in the description of the video.

To conclude the meeting, advisors were asked to reflect on their experience of being a part of this project, and were prompted with five questions: 1) What did you get out of participating? 2) Did it meet your expectations? 3) Did anything surprise you? 4) What have you learned? 5) What do you hope will result from your contributions? Advisors were given five minutes to reflect and put their responses to the questions anonymously on Jamboard with no prompting from the meeting facilitators. The Jamboard can be found in Figure 1.

## **2. Young Persons Reporting Guideline (YPRG) Workshops**

A series of two YPRG workshops were held in December 2023 – January 2024 and were focused on understanding what young people think should be reported in paediatric/child health RCT protocols and reports. Discussions during the workshops yielded potential reporting items for the SPIRIT-C 2025 and CONSORT-C guidelines. We engaged a wider group of young people across five countries: Canada, England, France, Scotland, and Spain through a series of two Young Person Reporting Guideline (YPRG) workshops. In total, 42 young people (ages 10-21 years) attended the first workshop, and 41 young people attended the second workshop. Specifics on the planning and composition of the YPRG workshops are elaborated below.

### **Workshop planning**

A “hub-and-spoke” working model was adapted for the planning and conduct of the workshops. The International Youth Involvement Steering Committee, and the YAG collaborated in the design and planning of activities for involving young people internationally in the SPIRIT | CONSORT-C project and formed the “hub”. The “spoke” comprised the different international YPAG groups from England, Scotland, Spain, France, and Canada. Each “spoke” conducted their own Young Person Reporting Guideline (YPRG) workshops with materials developed by the “hub”.

Consensus was met by all members of the International Youth Involvement Steering Committee to conduct two workshops. The first workshop was dedicated to introducing the project and thinking of information important for SPIRIT-C 2025, and the second workshop focusing on CONSORT-C 2025 and taking a deeper dive on any relevant topic points that came up during the first or second workshop. The steering committee discussed how best to involve young people during the workshops and how to meaningfully engage them in thinking of what important information should be reported in a trial protocol/report to come up with potential reporting items for the guidelines. The eYPAGnet youth facilitator experts shared their success with using personas within their YPAGs to foster meaningful discussion on other health research topics, as they put concepts into an understandable and relatable context for young individuals. Consensus was reached on developing multiple personas with varied health conditions, situations, and interventions. It was decided that the core team members (MS, AB, MO) would develop a first draft of the potential slide deck and seek feedback from the YAG on the proposed approach and the content of the materials, as described earlier.

After the YAG meeting, feedback was applied to the materials and then shared with the rest of the International Youth Involvement Steering Committee. Feedback points were discussed, and further discussions were held on workshop structure and how best to encourage young people to come up with reporting items during each workshop. The core team then applied all feedback and created a new version of the slide deck, materials, and evaluation forms. After a final round of review by all steering committee members, all materials were finalized; those who needed materials translated to Spanish or French did this with the help of their institution. Working plans were established on sharing through e-mail with all steering committee members to report on the individual YPAG workshops so that each group could learn from one another’s experience. Workshops were conducted either virtually or in person depending on the YPAG. An experienced youth facilitator (MP) was recruited to facilitate the workshops in Canada; workshops conducted with the established YPAG groups were led by their usual facilitator.

### **Identification of workshop attendees**

For established YPAGs, workshop attendees consisted of those that were already YPAG members. In Canada, a new group of young people (ages 12-18 years) were identified to take part in the workshops, as no central YPAG existed for the SPIRIT | CONSORT-C project. Members of the YAG were invited to attend the workshops; five out of six expressed interest in joining the workshops.

The project manager (AB) contacted several contacts affiliated with various YPAGs, Youth Advisory Committees, Patient Advisory Committees, and Youth Advisory Groups across Canada in November 2023 with a request to circulate information on the workshops and the expression of interest form to their groups. We focused on recruiting youth who were already involved with established YPAGs, Youth Advisory Group/Committee/Council/Panel, Youth Action Council, Youth Forum, Youth Research Advisory Panel/Group, or Patient Advisory Committees. In addition to the five YAG members who initially expressed interest, six more youth expressed interest by filling out the online interest form to join the workshops, bringing the total to 11 youth who were invited to attend the YPRG workshops in Canada.

### **Workshop #1: Project introduction and focus on SPIRIT-C 2025**

The first workshop was conducted during December 2023 and January 2024 and were scheduled by each group for a day and time that was suitable.

Though there were some variations across groups on how the workshops were conducted, all workshops used the same slide deck and structure. In brief, the first workshop started with an icebreaker, followed by introductory information, including project background and rationale, explanation of new concepts (e.g., research reporting guidelines, the need for paediatric/child health specific reporting guidelines, difference between a trial protocol and report), the final deliverable, and workshop goals. The participants were then introduced to RCT protocols from multiple perspectives: trial planning from the perspective of a trialist, and from the perspective of a youth who was looking to join a trial and was reading a trial protocol to learn more. This was done to prepare youth to think about what important information should be reported in a RCT protocol. Participants or YPAG members were then prompted to think about what information they would want to read about in a trial protocol before deciding to join an RCT, thinking about it from the perspective of themselves, or through the different scenarios and personas presented.

### **Workshop #2: Continuing the discussion and focus on CONSORT-C 2025**

After the first workshop, the second workshop was conducted during December 2023 and January 2024, scheduled independently by each group.

Similar to the first workshop, though there were some variations on the conduct of all the workshops, in summary, the second workshop started off with a recap of the first workshop and focused on RCT reports from the perspective of reporting the results of a trial. Trial reporting was presented from the perspective of a trialist and from the perspective of a young person who was reading a trial report in making healthcare decisions for themselves. The participants were then prompted on what information they would want to read about in a trial report before making decisions about their healthcare, thinking about it from their own perspective or through the personas that were presented.

At the end of the workshop, attendees were asked about their preferences of the name of the reporting guideline and were free to suggest potential name options.

### **Post-Workshop**

After the workshops, each group organized their workshop notes for review by the core project team. Core team members (AB, MO) thematically organized all notes from the workshop to identify new items that emerged from the discussion (Youth Generated items), and to see if the points brought up by the workshop attendees confirmed the importance of some of the items that are already part of the candidate item list (Youth Endorsed items). To close the loop on the workshops with the attendees, a “What we heard, what we did” document was developed (e.g., developed new items, or why new items couldn’t be developed from their points), and brief information on the next steps in the project, and when they could expect to see the final checklists.

## **3. Delphi study**

Young people (ages 19-24 years) with a) lived experience of participating in a paediatric trial; b) who read research and use trial results to make healthcare decisions for themselves; or c) have experience collaborating with researchers on a research team were invited to be a Delphi panellist. Four young people (ages 19-24 years; average: 21.5 years, all female) expressed interest and attended the Delphi Training Session and completed all three rounds of the Delphi study. Their backgrounds were diverse, ranging from lived experience of participating in a paediatric trial, experience working with researchers as part of a youth advisory group or as a family partner, or as a research team member conducting a paediatric trial. During the Delphi study, panellists were asked to rate the importance of including candidate reporting items in the final guidelines. Specifics about the Delphi study are published elsewhere (1, 2). All interested young people were invited to a one-hour virtual training session prior to the commencement of the Delphi Study, detailed below (Section B, 2).

# **C. Involvement of Family Caregivers**

## **1. Formation of the Family Caregiver Advisory Group (FCAG)**

To form the FCAG, recruitment materials were circulated through e-mail to the INFORM RARE Parent Advisory Group and CommuniKIDS Parent Advisors. The Patient Engagement Expert (MS) also reached out to their personal contacts and networks. In total, five family caregivers attended the FCAG onboarding session.

In total, four virtual FCAG meetings (1 hour each, with exception of the onboarding session) were held from October 2023 – November 2024. The project team communicated with the advisors through e-mail in between meetings.

### **Meeting #1 – Onboarding session**

Family caregivers who expressed interest in joining the FCAG were invited to a virtual onboarding session where they were introduced to the team, project, and what being an advisor would entail. Additionally, an overview of the Delphi study was presented that constituted a large part of their involvement in the project. The onboarding session was facilitated by members of the core project team (MS, AB, MO). At the end of the meeting, attendees were given the link to the evaluation form to provide feedback on the onboarding session and recruitment materials and were asked to also complete a post-onboarding interest form. All attendees expressed interest in being a part of the FCAG.

### **Meeting #2 – Advice on Delphi study materials**

Prior to the launch of the first Delphi round, we had a one-hour FCAG meeting to discuss what information is needed for the Delphi training session with other family caregivers, as well as feedback on the Delphi survey. Advisors shared input and feedback on slides that would be used during the training session, and provided suggestions on the wording, order of slides, and content. For the Delphi survey, advisors provided feedback on clarity and wording of the Delphi instructions, rating scale and colour codes, and explanation of the different candidate reporting item types. We ended the meeting with reflections on terms or concepts that need to be defined in the glossary and discussed how to motivate family caregivers to stay on for all three rounds of the Delphi.

### **Meeting #3 – Consensus meeting and E&E Prep**

Advisors were invited to attend the June 2024 Consensus Meeting, and out of five advisors, four indicated availability and interest in attending. A preparatory meeting was scheduled two days prior to the Consensus Meeting with all advisors. Prior to the meeting, we sent all Consensus Meeting attendees, including advisors, pre-meeting material (i.e., Consensus Meeting package, glossary, voting “cheat sheet”) for review.

At the preparatory meeting, we went over the Consensus Meeting purpose, agenda, process, and their role to prep them on what to expect and how they could meaningfully contribute. We also introduced and went over the sign-up and writing process of the Explanation and Elaboration (E&E) documents. Advisors were informed that signing up as a writer/reviewer for the E&E was optional, but as in the past, patient partners expressed interest in being a part of this process (3), so we offered them the opportunity to sign up as a writer/reviewer if interested. More details are below.

### **Meeting #4 – Knowledge translation and reflections**

In this final advisory group meeting, an overview of project progress was given. We also explained how knowledge translation was a key part of the project, and how it has been done in other similar projects in the past. We opened discussion to hear from them on what strategies would be effective (e.g., infographics, videos, utilizing networks of hospitals, social media, etc.). We also had time for everyone to share their reflections on project involvement. Part of the meeting was also dedicated to introducing a related project: the Template for Intervention Description and Replication (TIDieR) for Children and Adolescents (TIDieR-C). We explained what TIDieR-C is, why it’s needed, and how and why they should be involved, if it was of interest to them.

## **2. Delphi Study**

For the Delphi study, we aimed to include more family caregivers to capture a larger group of perspectives. Prior to the Delphi study, all interested family caregivers attended a training session (see below). In total, 10 family caregivers, including the five advisors, completed all three rounds of the Delphi study. Five (50%) of family caregivers have child(ren) who participated in a paediatric trial, one (10%) has child(ren) currently enrolled in a paediatric trial, and nearly all (90%) read trial results to make healthcare decisions for their child(ren). All panellists rated the importance of including candidate reporting items in the final checklists, and all young people and family caregivers who signed up completed all three rounds of the Delphi study. The specifics of the Delphi study are detailed in the statement papers, published elsewhere (1, 2).

### **Delphi Study Training**

To help in identifying family caregivers and young people (ages 19-24 years), throughout November 2023, a few FCAG advisors circulated information on the Delphi study through their networks and contacts to reach potential family caregivers (i.e., Canadian PKU and Allied Disorders (CanPKU+), Children’s Hospital of Eastern Ontario (CHEO) Research Family Leader Program, and the Youth Engagement in Research Instagram account (@youth_in_research)). Additionally, the project manager (AB) contacted several contacts connected with family caregivers and young people with relevant experience. In addition to the five FCAG members who signed up to be Delphi panellists, 10 family caregivers expressed interest, bringing the total to 15 family caregivers who were invited to attend the training. In total, we had 14 attendees across the two training sessions (10 family caregivers; 4 young people (ages 19-24 years)). All FCAG advisors attended the training session, though it was optional for them as the session contained information that they have already seen and advised on. The one-hour virtual Delphi training session was scheduled for January 2024 prior to the launch of the first Delphi round. As we were unable to identify one day and time that worked for everyone who expressed interest in attending the Delphi training session, we scheduled two times on the same day, one in the early afternoon and one in the evening. The training session was facilitated by members of the core project team (MS, AB, MO), and attendees were introduced to the team, project, what a Delphi study is, and what being a Delphi panellist would entail. Afterwards, attendees were asked to complete an evaluation survey on the training session and to confirm interest in being a Delphi panellist; all attendees subsequently signed up to be a Delphi panellist.

During the Delphi training session, we went over the purpose of the project, what reporting guidelines are, differences between trial protocols and reports, and why family caregiver and young people perspectives are important. We also explained what a Delphi study is, and how they have been used in reporting guideline development. Practical training included going over what each Delphi round would look like, along with instructions on how to complete the survey with screenshots of the survey. We also shared helpful tips, such as the ability to enable the text-to-speech functionality, how to download the full list of candidate items and glossary, as well as the ability to save and return later to the survey. We also left ample time for questions and emphasized that those with questions can reach out on their own time through email.

## **3. Consensus Meeting**

All family advisors were invited to attend the virtual Consensus Meeting in June 2024. The purpose of the Consensus Meeting was to discuss 13 reporting items that did not meet threshold for inclusion or exclusion during the Delphi study, and to introduce the group writing process of the E&E documents. All family advisors (n = 4) who attended completed the Delphi study and had a level of familiarity with the candidate reporting items. To enable them to participate to their fullest, a pre-consensus preparatory meeting was held with just the family advisors to explain the purpose of the Consensus Meeting and introduce the E&E writing process. Specifics of the Consensus Meeting are detailed in a separate publication (1, 2).

### **Pre-Consensus Preparatory Meeting (and E&E writing process training)**

To facilitate effective participation of family caregivers in the Consensus Meeting and the E&E writing process, we conducted a preparatory meeting to go over what the Consensus Meeting is, what to expect, and their role at the meeting. We showed an example of how the process would go when discussing a reporting item and set clear expectations on how they could participate. For example, we mentioned that family caregivers will be invited to share their thoughts first after an item was introduced, followed by input from other meeting attendees. We also set clear guidelines on time limits (i.e., 10 minutes per reporting item). Though the E&E writing process was going to be introduced to all attendees during the Consensus Meeting, we took time to explain the process to them, emphasizing that this was an opportunity that was available to them should they be interested in it. We explained how they could be involved, and in what ways they could contribute (writer and/or reviewer).

## **4. Explanation and Elaboration (E&E) Writing Process**

All family advisors were invited to contribute to the E&E documents as part of the writing/review team. Those who attended the Consensus Meeting (n = 4) signed up to write/review a combined total of 16 reporting items alongside 23 other writing team members over two months (July – August 2024). As writers, they contributed to the explanation text and identified good reporting examples for reporting items that they signed up for, and as a reviewer, they reviewed explanation texts and examples identified by other writing team members. Further details of the E&E group writing/review process are elaborated in the E&E papers (4, 5). Family caregiver advisors were also invited to contribute to the generation of Paediatric Considerations for 12 items in the Template for Intervention Description and Replication (TIDieR) guidelines as part of the efforts in developing the TIDieR-Children and Adolescent (TIDieR-C) guidelines through a virtual workshop. Details of the development of TIDieR-C are published separately (6).

# **D. Qualitative Feedback from Evaluation Surveys, by Key Partner Group**

## **1. Feedback from Youth Advisory Group**

| **Feedback after Youth Advisory Group Meeting #1 – Onboarding Session** |
| --- |
| *Is there anything else you’d like to tell us about the onboarding session?*   - At one point [name redacted] talked about understanding the clinical trials we went through, but on the slideshow it mentioned other ways we could be a part of the advisory group even if we were not in a clinical trial. So I am just wondering if being a participant in a clinical trial is mandatory because for example I am not, however I have been a participant of research studies and have read studies alongside my parents to make informed decisions about my health. If you could clear up information on this that would be great thank you. - There's nothing else I'd like to say :)   *General comments on recruitment flyer – is there anything else you’d like to tell us about the flyer?*   - You may want to choose colours with greater readability/contrast as grey against muted blue may be hard to read, especially if someone has colour deficient vision or visual improvement - Nope, overall a good flyer. - No   *General comments on the information sheet – is there anything else you’d like to tell us about the information sheet?*   - I think there should have been an age range of certain jobs given to said age in order to know what teens and adults would be able to do or how their jobs would differ - Nope once again a good sheet. - No |
| **Feedback after Youth Advisory Group Meeting #2 – Workshop Content** |
| *No textual comments were left by the advisors* |
| **Feedback after Youth Advisory Group Meeting #3 – Knowledge Translation, Reflections** |
| *Is there anything else you'd like to tell us about your experience with this project?*   - this was a fun way to help others and would love to continue help in this field - thank you for the opportunity to participate :) - I’m always happy to share my experience, knowledge and opinion, especially when I am heard and listened. This project let me do just that and I see my input being used in the project. - Great experience! - No thanks :) |

## **2. Feedback from Young Person Reporting Guideline Workshops**

| **Feedback after Young Person Reporting Guideline Workshop #1** |
| --- |
| *Is there anything we can do to make workshop #2 better?*  Canada workshop (n = 7)   - I think it went very well! - I mean I'd love if more people could speak up, but there's not a whole lot to be done about it - Spend more time explaining instead of speeding through things - No   England workshop (n = 2)   - Mentimeter may be more convenient for collaborative discussion as opposed to Jamboard - N/A   Spain workshop (n = 6)   - I felt that I was lacking some information about the target audience of SPIRIT.   *Is there anything else you’d like to share with us?*  Canada workshop (n = 7)   - I really enjoyed the workshop and thought that it was a great and useful conversation. I found the explanations very clear, and the different methods of discussion were great! (chat, Google Jamboard, talking). - I have [redacted as potentially identifying]. I think I would do better in a one-on-one session instead of a group workshop. |
| **Feedback after Young Person Reporting Guideline Workshop #2** |
| *Is* *there anything else you’d like to tell us about today’s workshop?*  Canada workshop (n = 6)   - Thank you! - Im excited to see how this goes! Always happy to participate :) - No   England workshop (n = 4)   - Thank you for your time and the opportunity - N/a |

Note: Not all attendees of the YPRG workshops provided feedback

## **3. Feedback from Family Caregiver Advisory Group**

| **Feedback after Family Caregiver Advisory Group Meeting #1 – Onboarding Session** |
| --- |
| *What were the strengths of the onboarding session?*   - love multiple presenters. helps keep attention at the end of a long day. - Easily digestible format and presentation. Speakers changing for different slides prevented monotony - Relevant and accessible information. The presence of a facilitator. Charismatic presenter. A very interesting project.   *What could be improved about the onboarding session?*   - it felt a little long, some repetitive slides/information but I think it was valuable for this entering for the first time. - There is always room for improvement but the team is already inviting us to evaluate with good tools so it should continue like this.   *What else would you like us to know about your experience with the onboarding session?*   - excited to move forward. also excited to help learn about E&E and paper writing (if considered) for my own personal growth and development as well. - Our experience with clinical trials and access to medicine has led us to collaborate with industry and policy makers. I hope that the guideline will be used by all stakeholders.   *General comments on recruitment flyer – is there anything else you’d like to tell us about the flyer?*   - it was very heavy in information. I feel in the why you should participate...the first two statements would do better if switched order - Initially looked very wordy and cramped in terms of content in a tiny space. - Maybe fewer words, more pictures?   *General comments on the information sheet – is there anything else you’d like to tell us about the information sheet?*   - under why are they called spirit and consort...you could maybe have mentioned the name is coming from the pre-existing "adult" version, which were named as an acronyms of (still not sure what the original name came from... Google will tell me in a minute) this goal of adapting these documents to the pediatric community may have encouraged others to attend? |
| **Feedback after Family Caregiver Advisory Group Meeting #2 – Delphi Feedback** |
| *What were the strengths of today’s advisory group meeting?*   - great meeting. light but informative. - It is obvious that the feedback was taken seriously. Everybody seemed very comfortable to speak. - Seeing how feedback was put to use. - It was really great to meet everyone and get to know them better. The informal environment allowed for openness and comfort in sharing. The team was very receptive to our feedback and ideas for change, this was really nice. - The strengths is the people who were present. Good energy. Dynamic presentation. Listening, sharing opinions and having fun. Only pluses   *What could be improved?*   - While it was optional to read slides beforehand, it helps some of us to think and be better prepared. I would have liked to have a bit more time to look at the slides. - Maybe add a little more time at the end for folks who want to stay on a linger a little but can 'end the meeting' for those who need to run. There might have been a bit too much material to cover in one hour but I appreciate [name redacted] trying to keep us mostly on time, lol! |
| **Feedback after Family Caregiver Advisory Group Meeting #3 – Prep for Consensus Meeting and E&E** |
| *What were the strengths of today’s meeting?*   - Well laid out slides that clearly described the process, respectful discussion, lots of time for questions. The consensus meeting will be easier for us because of this pre-meeting. Lots of practical details were shared e.g. 1-2 sentences, not more then one minute, we only have ten minutes to discuss each item   *What could be improved?*   - Some of us had technical issues, but that is not a reflection of how well this meeting was planned. It is clear that you are equipping us so that we can do our best with this process. |
| **Feedback after Family Caregiver Advisory Group Meeting #4 – KT, Reflections, and TIDieR-C Info** |
| *Is there anything else you’d like to tell us about your experience with the SPIRIT \| CONSORT-C Project?*   - It was such a fabulous learning experience, and I felt part of the wonderful team   *Is there anything else you’d like to tell us about your experience with the TIDieR-C information session?*   - Excited to learn and participate |

## **4. Feedback from Delphi Panellists**

| **Feedback after Delphi Training Session** |
| --- |
| *What were the strengths of the Delphi training session?*   - Very nice to have the slides to review beforehand; information was clear and concise. - Clear slides, ability to ask questions and get responses, multiple ways of interacting, within the scheduled time, no expectation of previewing slides, but we had in case we want to preview or review, timely reminder of the session (sometimes reminders come too early - more than a day or two before or too late - just as a session is starting) - It was well done, nice slides, variety of speakers. I liked the set up of the meeting, it was easy to feel welcomed and included - Very glad that you provided this training session. I found the slide about the overview of the study and where we are helpful. - Instructors were able to answer all questions and explain every aspect of the study in detail. This made participating in the study less intimidating. - It was clear and the talking about the session was specific. I liked that lots of opportunity for questions was provided. - I appreciate the opportunity to connect with the various stakeholders, learning a little more about them each time increases my feeling of belonging to the group and the project. This enhances the fact that I also learn things and feel useful. The abilities of stakeholders to create a safe space to express questions and opinions. Information provided in advance. - Very welcoming team! Plenty of time was given to ask questions and at many points. Good pace (not to fast, not to slow) Very clear presentation - answered all my questions along the way - The slideshow presentation was visually appealing and organized in a way that clearly demonstrates the key information of the study. The speakers identified the plan clearly and efficiently. - time, detail of info, format   *What could be improved about the Delphi training session?*   - More of an introduction in terms of people's roles - the titles/roles of the 6 people highlighted on the beginning slide, who the Parent Advisors were, etc. - It was an excellent session. - This is already such a vast improvement from the last Delphi I tried. I didn't feel connected or valued on that one, and it was so much work, I dropped out by the final round. I feel very invested in this project and that my viewpoint is heard and valued - Would have liked to have seen the children's workshops listed in the overview slide and also the delphi training session to get a sense of everything that is happening and when. Would have like to have gotten a definition upfront about Delphi and even what Candidate items are. I have never heard the term candidate item and am still a bit unclear about what it is. - Typically this is where I mention that it is helpful to receive the slide deck ahead of the presentation so that I can form my questions ahead of time; however, the trainers provided the slide deck ahead of time! very happy about that as it made following along that much easier. - keep up the good work. - By continuing to listen and implement the diverse voices of patients and their caregivers, improvement should be continual. The rhythm is good - More interactive - could've done introductions verbally instead of chat but this is very minor. - N/A - describing the timeline as a whole for the entire project earlier in the presentation and then walk through what happens after this first meeting   *What else would you like us to know about your experience with the onboarding session?*   - it might have been useful to provide the slides and then allow people to opt out of the training session. Having done Delphi studies before, I probably did not need to attend this session and it did take away from my work day (although I always enjoy seeing those familiar faces!) - Great experience other than dealing with a new health issue that affected my attention level. - Many thanks, it's so well organized and been ++ enjoyable journey so far. - It would be very helpful to have gotten the overview slide when this meeting was initially being set up. I am also on the advisory group so I need to wrap my head around what each meeting is for. NOTE: prior to the session I did not have a clear understanding about the purpose of the meeting, but now that the meeting has happened I understand much better. - I enjoyed having multiple instructors/presenters providing their input, and being able to see the presenters on camera. I appreciated the multiple opportunities to ask questions and receive further explanations. - great idea for the ice breaker to use the chat. - Juste vous dire merci et signifier ma gratitude pour cette opportunité. - So far so good :) - I enjoyed it thoroughly and gained new insights! - It was a great presentation. |
| **Feedback after Delphi Study (Survey)** |
| *Lastly, if you have any comments about the Round 3 survey, please leave them here:*   - I really liked the way you set up the surveys. It was very helpful to have the graphs and also be reminded of my previous scores. I liked how you let me know how long it would take to complete, so I was able to plan accordingly so I didn't feel rushed. I liked seeing the summary of feedback on the items. I know there was a lot of text to review, but with the great training, and layout I found it smooth and manageable. It was a joy to be a part of this project, it was a very positive experience and I learned a lot about clinical trials in the process. Thank you :) - I found the time estimates to be a little short especially if you were to refer back to results from earlier rounds. - Thank you so very much, I greatly appreciated being a part of the delphi study:) - Thank you to the team for helping me further my knowledge into this research tool. Your guidance provided enough materials to make me feel like my contributions would add value even though I was new to this tool. Your support is truly appreciated through this process! - Thank you for this great experience! - It was a first experience for me, I was perhaps a little shy in expressing my opinions in writing but the voting grid allowed me to express myself. The comments and summary made by the team were very useful and appreciated. The work of your team is very relevant and of great value. |
| **Post-Delphi Debrief/Focus Group (Note: parts of the feedback below was transcribed live, and also supplemented by typed input in the chat)** |
| *How helpful did you find the training session in January in preparing you for the Delphi study? What worked well and what could be improved about the training session?*   - **Panellist 1:** From my point of view the training session was totally necessary. I would not have understood how to do the first survey without it. Before this exercise the word ‘Delphi’ was quite daunting for me. After the training, I had more confidence - **Panellist 2:** It was helpful but I still was a bit confused when it came to the first session- after that I understood. For the training a lot of pieces were explained but maybe having a visual map of all the moving pieces and highlighting what part we were being involved with directly/ what the steps were for us - **Panellist 3:** Yes it was I am not sure I would change anything yes to necessary - **Panellist 4:** I think it would be helpful to have even more training on the Delphi process in general, like something all parent-partners should take. The Delphi process comes up a lot in research. (**Panellist 1** agreed to attend more training) - **Panellist 5:** Needed that training as I would have had no clue otherwise what to do - **Panellist 6:** “I appreciated having the visuals that we could refer back to and the definitions and what exactly we were looking for. And then we were able to go to this session and ask questions and do whatever we needed to do.” - **Panellist 7**: yes, very helpful. have done Delphi's before but more examples would have been helpful - **Panellist 8:** I would’ve liked more training as well, and I like the idea above about visuals. I think more hands-on practice during the training (i.e. practice items) would have also been helpful   *What did you think about the full glossary and the glossary box in the Delphi?*   - **Panellist 9:** “I found it helpful, for sure. I thought the language was good.” - **Panellist 2:** No negative feedback on the glossary - **Panellist 10:** The glossary was helpful - **Panellist 8:** “I also agree that it was helpful. I come from some research background. So a lot of the terms were terms I had known already. But I think reading them and kinda like knowing that the definitions were clear. Is good feedback like that so you can know that like kinda it would be helpful, for others like the terms were very clear, and the definitions as well.” - **Panellist 1:** I have been in many meetings where the scientists use all the terms very freely and I often feel intimidated. The glossary is helpful and I like how I can read it on my own time. - **Panellist 7:** I liked having the glossary definitions right in the survey question (where applicable). (**Panellist 1**, **Panellist 5**, and **Panellist 11** agreed)   *How was the length of the Delphi study? Did you think that it was a worthwhile use of your time?*   - **Panellist 9:** I guess that kind of goes back to you guys in the question was, did you get the results that you were looking for from the time we put in. - **Panellist 1:** The first survey was very long and I wasn’t quite prepared for that. Would have liked a bit more of a heads up. The other 2 went more smoothly because a) I was prepared b) I felt more confident - **Panellist 6:** How long did it take people to complete the Delphi? Did it reflect the time that was expected? - **Panellist 8:** I definitely think it was worth the time - I feel like I also learned so much being part of the process. Just as a reminder, I think it said something like 40 minutes to complete, I think that that was kind of the length of time it take. It took me, maybe the first one slightly longer, but I would say that was an accurate estimate. - **Panellist 1:** “Usually with surveys, they'll say, ‘Oh, it'll take you half an hour to take to do.’ But it actually takes like 15 min. So it was like a real 40 min. So I don't think I was prepared for that, because lots of surveys like, oh, it's gonna take 40 min, but it really takes 20 min. So it was accurate. But I wasn’t quite prepared, based on history of taking surveys.” - **Panellist 10:** “Yeah, I kind of agree what everyone was saying. I think the first one took so much longer to, though which kind of corresponds back to the training, as I was trying to like, understand the differences and the processes and all of that, so I reread a bunch of stuff as I was going through so obviously that added like a lot more time. And then it was smooth sailing from there.” - **Panellist 2:** The first one took me longer than 40 because I was reading everything and getting to know the format (**Panellist 6** agrees) - **Panellist 11:** Agreed, the first one took longer just because I had to adjust to the amount of reading and process. But I did like the text that was included. It orientated me for the rounds.   *Did you feel that your input/the input of youth, young people, and other family caregivers made an impact on the Delphi surveys? Why/why not?*   - **Panellist 6:** Really appreciated the input from the young people. - **Panellist 2:** I liked seeing that - **Panellist 5:** They def did - **Panellist 1:** Liked seeing the feedback from the youth - **Panellist 4:** It was good to see the youth perspective - **Panellist 10:** Nice to see it - **Panellist 2:** I thought that some points that were brought up were unique and surprised it was not considered before (**Panellist 1** agreed) - **Panellist 6:** For me, it showed that you were listening and helped with trust.   *Do you think it’s important that we included youth, young people, and family caregivers? Why or why not?*   - **Panellist 6** and **Panellist 2** agrees and absolutely thinks so - **Panellist 1:** It’s really great to include youth perspectives - it bring a new perspective that is needed. - **Panellist 3:** “I think it really helped with giving like a holistic perspective on it. And sometimes, even though you might have a certain opinion about something. Not that you need it validated. But it's like, Oh, okay, like a little bit of a check in, too. And looking at the responses. Right? That's what I liked about it.” - **Panellist 1 (in response to Panellist 3):** “Yeah I agree. I do like the validating aspect. But I think what surprises me is that, I’ve done projects with my daughter sometimes. And it’s like we’re living the same life through the same experiences. But I’ll say something, and she’ll come up with things that I would have never have thought about. But then I’m like, Oh yeah. And it just really shows that, you know, maybe as I age, I just have a way, different perspective. And it’s really refreshing, and I think totally important to just bring those youth voices, because as you age, and as you’re an adult, you kind of lose that.”   *How did your experience of being on the Delphi study change as you progressed through the rounds? What sparked the changes?*   - **Panellist 1:** I felt more confident in the process after each round (**Panellist 3** and **Panellist 4** agreed) - **Panellist 7:** the first one was definitely the longest and the most confusing - subsequent rounds were easier because I was more familiar with it and they were less time consuming - **Panellist 5:** I also liked the fact that it showed me how I had voted before. (**Panellist 1** and **Panellist 2** agreed) - **Panellist 2:** I was interested to see what items would be brought back - **Panellist 6:** “Again, I appreciated all the different perspectives, and I want to circle back to when you did the training. And how you stress that one viewpoint in the first round doesn't mean you have to have the similar viewpoint, that you can change your mind. And as we went through I was like a bit more open minded about things, and definitely changed a lot of my ratings for at least some of my ratings. I shouldn't say a lot I don't know, but I did change some of my ratings.” - **Panellist 10:** I didn’t love seeing what I did before too much and others as I found it persuaded my responses seeing what I voted compared to others. - **Panellist 8:** “I appreciated the feedback both with like getting my responses and also others. Just because I think it helps with critical thinking. And kind of understanding that there are different ways to go about research and kind of different perspectives to consider. As we like to see with holistic care. So I think I think, yeah, definitely developed my critical thinking. And made me made me kind of excited each time to go through the rounds.” - **Panellist 9:** I like seeing it, but looked after choosing my new score. Was interesting to see what I was swayed by - **Panellist 6:** Absolutely helped to develop critical thinking. - **Panellist 4:** I was sometimes surprised how I changed answers from round to round, most of them stayed the same, but I did change some. (**Panellist 6** agreed)   *What do you think about the number of panellists responding from the perspective of a young person or family caregiver in the Delphi study?*   - **Panellist 6:** Liked the variety of the panellists, but would have like to see more youth and caregivers. - **Panellist 2:** I think the protocol inclusion I understand the number of having more academics- but for the actual paper I think more audience friendly should be considered - **Panellist 1:** Would have liked to see higher than 10%. Would like at least 20% of youth and caregivers - **Panellist 6:** Did you have a goal in mind as to how many people you wanted? - **Panellist 8:** The perspectives can’t be generalized across all young people and families - really small sample - **Panellist 2:** “I definitely agree with what was just said as well, and I thought it was interesting, even the way I was feeling, answering the questions between the protocol. I forget what the 2 parts were, but one of it was for the protocol rating, and the other one was for the actual inclusion in the paper. Because I do think the audience and the families are more considering the papers. and I get there has to be that science element from more, maybe, of the academics, for all of the other details in the in the protocol. But for the actual presentation of the paper, I do think more family voices, that would be good.” - **Panellist 1:** “Yeah, I just wanted to say that as I was going through the surveys I sort of I I sensed that it was a little bit sort of science researcher heavy, and that, you know, this lived experience group was smaller, and that kind of drove me to like stick with it, even though the first one was like pretty, daunting, and very long longer than I expected. I was like, no, I gotta see this through the end, just because I I wanted that voice to be heard.” - **Panellist 3:** “Sometimes there’s not enough general information right about, you know your kid’s diagnoses. So then that’s where you end up going for your information right? And if it’s all just medical speak, that’s great because it kind of gives you that piece. But then it’s also really really important to hear about the families, because you don’t have that information anywhere else.”   *What did you get out of participating in this project so far? Was this in line with your expectations?*   - **Panellist 6:** Really appreciated the timely compensation and the choice with the Everything card. (**Panellist 2** and **Panellist 1** agreed). Yes and you were clear about the timeline and actually pretty good about actually sticking to it (**Panellist 10** and **Panellist 5** agreed) - **Panellist 1:** Yes, I found the exercise valuable and now I know what a Delphi survey is! (**Panellist 10** and **Panellist 6** agreed)   *What did you think about communications with the research team during this project so far? What has worked well, what could be improved?*   - **Panellist 8:** The reminders were super helpful for me - **Panellist 10:** Communication was well done! - **Panellist 2:** Yes communication was helpful and I needed the reminders for sure - **Panellist 1:** Communication was great - **Panellist 3:** [Point person] was fantastic (**Panellist 1**, **Panellist 9**, **Panellist 5**, **Panellist 8**, **Panellist 6**, **Panellist 4**, **Panellist 11**, **Panellist 2** agreed) - **Panellist 6:** Communication was good. Also with the timeline, there was a good amount of time complete each round so we could fit around our schedules. (**Panellist 1**, **Panellist 2**, **Panellist 4** agreed)   *What difficulties have you experienced as you were participating in this project so far? How were they addressed or worked through?*   - **Panellist 10:** No issues getting in. Was nice we could go in and out. - **Panellist 2:** Yes because it revisits topics to come to a team decision   *What advice would you have for other researchers working on methodological reporting guideline projects who wish to involve young people or family caregivers?*   - **Panellist 3:** “Well make sure you have an [point person] – because parents are gonna ask lots of questions, right? And like, I know for myself, kids in school. There's all that going on, you know, there's lots of layers right? So then sometimes you're so caught up in that. And then you're like, Okay, yes, I'm involved in this. and sometimes you feel like there's a gap. So you might go back and say, Hey, you just have questions right? So I think I would have for me, I just think, expect lots of questions and be patient.” - **Panellist 1:** “Role of [point person] and [patient engagement expert] – both are completely necessary in a project like this. I think I was in another meeting a week ago, and they were embarking on. They wanted to have people with lived experience to go through the Delphi process. and I had brought up. I said, Well, I just finish this thing, and you know there was a training session, and there's a you know, a parent person. And then there's the Coordinator that you can ask questions. And they hadn't even thought of anything like that, and I just think I would I would have dropped out like a long time ago if those roles were not embedded into this project.” - **Panellist 11:** Having a point person was key. Having [point person] made it more personalized, and helped me to buy in and feel like I was making a meaningful contribution. The updates and prompts were perfect. I really felt heard and seen. (**Panellist 6** agreed) - **Panellist 2**: “But the I think one thing that is really important in this, and it's different than just a single feedback session. For example, if you were to just show us all the questions, and then we just gave feedback that one time I think it was really important, and got like real answers from people when you revisit these topics. When you see them again, and like it makes you think more about it than just. You say something one time, and it's taken down. So I think the process as a whole is really good for that.” - **Panellist 1:** Having the opportunity to give feedback via this session is great - **Panellist 6:** Budget. Be clear about expectations. Be sure that the young researcher is familiar with the process themselves and has some training in patient engagement - **Panellist 5:** I felt like my inputs were having an impact. So it was very satisfying being part of this. |

## **5. Feedback after Consensus Meeting**

| **Feedback after Consensus Meeting – Family Advisors** |
| --- |
| *Participating in this Consensus Meeting allowed me to:*   - Broaden my horizons in how I can help rare disease. Educate myself about this process as well as new terms - Learn about clinical trials, make connections, and contribute my lived experience. - Learn from others from around the world and consider their perspectives, follow through on the rest of the process that was started with the Delphi, express my thoughts (and even change them) on the various items, etc.   *Challenges I experienced were:*   - All resolved by the fantastic team! [Names redacted] etc were all wonderful in anticipating our needs and providing what was needed. - The polls were a bit confusing at times. The meeting felt a bit long, but I think the time was well managed. I felt more comfortable writing in the chat. - I was mostly fine, but should have prepared better for the breaks. Once in a while, I had to consider the language used around some of the items. - it was my first time so i was unsure how it would all work, but [point person] was great and everyone extremely helpful. it was easy to follow   *Lastly, if you have other comments about the Consensus Meeting, please leave them here:*   - Thank you for allowing me (us) to participate - Thank you it was a great experience and all the items being reviewed were in clear language, so I never had to answer unsure :) Well done! - I really appreciated that [name redacted] told us she was there for support and also that some really kept redirecting us back to the input from the youth. It would have been great to have youth voices present. - thank you for all your work and leadership on this! |

## **6. Feedback after E&E writing process**

| **Feedback after E&E Writing Process – Family Advisors** |
| --- |
| *How easy was it to contribute to the E&E documents?*   - It was a lot more difficult than I expected. It also took more time than I realized. - The process was well laid out overall. The most difficult part was not communicating effectively in team. The process could have benefited from a request to meet as a writing team prior to starting the writing process so we could understand strengths and plan together. It may have also benefited from having separate documents for each item to work in instead of the very large document. - My shyness, lack of experience and confidence may have limited my contribution. An icebreaker activity or a team meeting with my writing and editing partners could have given me more confidence.   *How understandable was the process in contributing to the E&E documents?*   - The documents were helpful, though I did have to reach out for further clarification. - The process was laid out very well overall. It might have benefited from a separate example of an E&E - most people might have been familiar but not all. - Very clear, with all the tools, support and time to understand the procedure, the steps, the objectives.   *How much did you enjoy being part of the E&E writing team?*   - I still very much enjoyed the process, learning and collaboration. - I have put neutral here as there were some very good things about being part of a team. There was one member who was very good to obtain relevant references if I could not access them and there was another member that stepped into a void when little had been done on an item. This process through me back to a time where I was a partner and all was going well and then the researcher I was working with just stopped communicating - I now know it was an overwhelming time for [redacted] with lots going on, but it was very strange at the time and left me doing work that I was not originally supposed to do. It took longer than it should have and I had my own health issues to contend with but felt obligated to complete the project. Although I learned a lot and the researcher and I get along very well and have since interacted, it was difficult. I was hoping to meet deadlines, but set up documents after not hearing much from other team members. I tried to leave space for others to contribute, but it simply did not happen with one team member with the exception of occasional responses to E-mails and then silence. It would have been nice to just have a "I don’t have capacity right now".   *Was the amount of time it took to contribute to the E&E documents acceptable?*   - It took several hours and alot of coordinating. At times I felt a bit lost, and a team meeting with the working group would have been helpful, but I was already really busy and overwhelmed with some family matters so I didn't offer to organize. I did think about backing out a few times, but glad I stuck with it, but I do hope my team felt I brought something to the table. - The original timeline was too short and while [name redacted] very much deserves vacation time, it would have been great to be redirected to another person to ask questions during her absence which occurred near the beginning of the writing period - I find that I have read a lot (personal motivation), thought a lot, in relation to what I have written or interesting documents that I would have liked to share. This helped me to understand more deeply, not sure if I have managed to summarize my thoughts in a complete and clear way so that it is usable.   *How would you rate your overall satisfaction with being part of the E&E writing team?*   - Mostly because I felt bad that I wasn't doing enough, or not knowledgeable enough to contribute with confidence. - I am proud of what I have contributed, but wish that we could have worked together more effectively as teams. I have appreciation for the supportive team members, those that actually contributed to the writing process, but wish we had communicated better - I feel like I could have contributed more but I am confident in the process so that the project leaders will make the most of my modest contribution. A positive enough experience to want to do it again with the conviction that it is important to participate and that I can do better [now] I lived this experience.   *How helpful was the preparatory meeting prior to the Consensus Meeting to contribute to the E&E documents?*   - So glad we had that! - I think having a shared example here might have been beneficial. I went an looked at another E&E, but some may not have.   *I understood the objective(s) of the E&E documents*  *I understood how I could contribute to the E&E documents*   - I know how but I'm still developing the skills to do it in a meaningful way.   *I received sufficient explanation about how to contribute to the E&E documents*   - Have the opportunity to set an example as a team. A practice   *Being part of the E&E writing team was worth my time*   - It's important work and it was very rewarding to work on. - I agree [that being part of the E&E writing team was worth my time], even though I spent a lot more time than expected. It was worth my time because it aligned with some of my goals as a patient partner. I learned new skills and being part of this has a potential to contribute to better clinical trial reporting which in turn could improve the way paediatric clinical trials are done.   *I contributed by providing my perspective*   - Some of the examples I shared come directly from my perspective as a caregiver. some came from references. I have been of a clinical trial that we did not complete because of the way it was done (long story) and I believe that it could have been improved greatly. I also understand what my children and our family might need to participate.   *My workload as part of the E&E writing team was manageable*   - This was part me (really desiring to contribute effectively) and part the team (one of the three teams in particular). I found a number of examples for my writing items (and one for one on which I was a reader). This along with providing perspective after other members had some basic writing done seems to me to be an appropriate amount of work. However, I ended up reading a number of references and doing much more writing than I expected.   *I received sufficient support to contribute to the E&E documents (for example, training, resources, support from team members/research team)*   - Agree that [name redacted] provided wonderful support. The premeeting was helpful and hearing about it again on consensus day was great. Did not feel as supported by other team members (two were awesome, others not so much).   *Any concerns I had were addressed*   - [Name redacted] effectively addressed some concerns.   *Fellow members of the writing team(s) was (were) open to receiving my views*   - Some were.   *I was offered sufficient recognition for my contributions*   - I am hoping to have authorship from this. I also appreciate the honorarium and I knew the expectations starting the process so understood that I was ending up volunteering a fair bit of time. I was thanked several times.   *I enjoyed being part of the E&E writing team*   - I enjoyed learning about the process. I think if we had had team meetings at the beginning, learned each team members perspective for each item and "assigned" tasks, I could have enjoyed this more. I was also exposed to some materials that I might not have otherwise seen that are interesting too.   *I was able to contribute to the E&E documents*  *I was comfortable in contributing to the E&E documents*  *I think the input I provided in the E&E documents will make a difference to the development of reporting guidelines*  *Participating in the E&E writing process allowed me to:*   - to learn more about trials and bring my own values as a PWLE and also as a professional - learn new things from others and the materials; practice writing skills; use my searching skills effectively (to look for examples); learn more about what I value in teamwork - Learn new things, deepen my knowledge. Gain confidence. Discover and understand the importance of this tool. The importance of collaborating and bringing the point of view of patients and caregivers. Make me want to continue my involvement and promote it to other patients and caregivers.   *Challenges I experienced were:*   - finding examples for the guide. - timing - while I was keen to start, very little was done until after the initial deadline - Each item is so important, so many angles to cover. I always feel that one paragraph, one example will not be enough. I know that researchers and professionals already have a good prior knowledge on these elements but how to bring others to the second level. It remains superficial. Is there another level of information after the E&E?   *Lastly, if you have any other comments about the E&E writing process, please leave them here:*   - I would have valued a check in meeting for those having trouble and trouble shooting a bit together. I had a few emails but an optional group call with the leads would have been a great addition. - I have said most of them already. Things that might have been beneficial: 1) separate documents for the items already setup; 2) direction to have a team meeting or at least do E-mail intros with name, perspective bring to the project, and what role; 3) resource folder for protocols |

# **E. Quantitative Feedback from Evaluation Surveys: Heatmaps**

## **1. Feedback from Youth Advisory Group**

| **Administered scale - Time in project (number of feedback received)** | **+++** | **++** | **+/–** | **– –** | **– – –** |
| --- | --- | --- | --- | --- | --- |
| **Modified PPEET – After Meeting #1 (Onboarding)**  Response rate 100% (n = 6) | | | | | |
| Easy to attend | 4 | 1 | 1 |  |  |
| Clear and understandable | 3 | 3 |  |  |  |
| Helped me understand the purpose of the project | 4 | 2 |  |  |  |
| Learned how I could be involved in improving clinical trial reporting | 3 | 3 |  |  |  |
| Understand different ways I could be involved in this project |  | 6 |  |  |  |
| Enjoyed the onboarding session | 2 | 3 | 1 |  |  |
| Provided proper supports to join (e.g., time of the session, technical support) | 3 | 3 |  |  |  |
| Good use of my time | 3 | 2 | 1 |  |  |
| Satisfied with the session | 3 | 2 | 1 |  |  |
| **Modified PPEET – After Meeting #2 (Workshop Content)**  Response rate 100% (n = 5) | | | | | |
| Easy to attend | 1 | 3 | 1 |  |  |
| Clear and understandable | 2 | 3 |  |  |  |
| Understood the purpose of the meeting | 3 | 2 |  |  |  |
| Enough information so I could contribute to the discussion | 2 | 3 |  |  |  |
| Express my views freely | 2 | 2 | 1 |  |  |
| Feel confident that my input will be used by the research team | 1 | 4 |  |  |  |
| Provided proper supports to join (e.g., time of the session, technical support) | 1 | 3 | 1 |  |  |
| Good use of my time | 2 | 3 |  |  |  |
| Satisfied with the meeting | 1 | 4 |  |  |  |
| **Modified PPEET – After Meeting #3 (Knowledge Translation, Reflections)**  Response rate 100% (n = 5) | | | | | |
| Easy to attend | 3 | 2 |  |  |  |
| Provided proper supports to join (e.g., time of the session, technical support) | 3 | 2 | 1 |  |  |
| Clear and understandable | 3 | 2 |  |  |  |
| Understood the purpose of the meeting | 3 | 2 |  |  |  |
| Enough information to contribute to the discussion | 3 | 2 |  |  |  |
| Express my views freely | 3 | 2 |  |  |  |
| Feel confident that my input will be used by the research team | 4 | 1 |  |  |  |
| Good use of my time | 4 | 1 |  |  |  |
| Satisfied with the meeting | 4 | 1 |  |  |  |
| **Modified PEIRS – After Meeting #3 (Knowledge Translation, Reflections)** Response rate 100% (n = 5) |  |  |  |  |  |
| Understood the objective(s) of the project | 4 | 1 |  |  |  |
| Sufficient support to contribute to the project | 4 | 1 |  |  |  |
| Understood how to contribute to the project | 4 | 1 |  |  |  |
| Communication with research team clear | 4 | 1 |  |  |  |
| Being part of the Youth Advisory Group was worth my time | 5 |  |  |  |  |
| Contributed by providing my perspective | 4 | 1 |  |  |  |
| Any concerns were addressed | 3 | 2 |  |  |  |
| Research team was open to receiving my views | 5 |  |  |  |  |
| Offered sufficient recognition | 4 | 1 |  |  |  |
| Enjoyed being part of the Youth Advisory Group | 5 |  |  |  |  |

Questionnaire wording has been abbreviated for clarity and conciseness; Response formats are represented as +++ for strongly agree, ++ for agree, +/- for neutral, - - for disagree, and - - - for strongly disagree

## **2. Feedback from Young Person Reporting Guideline Workshops**

| **Administered scale - Time in project (number of feedback received)** | **+++** | **++** | **+/–** | **– –** | **– – –** |
| --- | --- | --- | --- | --- | --- |
| **Modified PPEET – After Workshop #1** (n = 15 \| Canada response rate 100% (n = 7); England response rate 20% (n = 2); Spain response rate 60% (n = 6)) | | | | | |
| Easy to attend | 7 | 7 | 1 |  |  |
| Clear and understandable | 8 | 7 |  |  |  |
| Understood the purpose of the project | 8 | 7 |  |  |  |
| Enough information so I could contribute to the discussion | 7 | 7 | 1 |  |  |
| Express my views freely | 9 | 5 | 1 |  |  |
| Provided proper supports to join (e.g., time of the session, technical support) | 8 | 6 | 1 |  |  |
| Good use of my time | 10 | 5 |  |  |  |
| Enjoyed the workshop | 8 | 6 | 1 |  |  |
| **Modified PPEET – After Workshop #2** (n = 16 \| Canada response rate 100% (n = 6); England response rate 40% (n = 4); Spain response rate 60% (n = 6)) | | | | | |
| Easy to attend | 10 | 6 |  |  |  |
| Explanations prepared me to discuss reporting in trial reports | 5 | 11 |  |  |  |
| Understood the purpose of today’s workshop | 11 | 5 |  |  |  |
| Enough information so I could contribute to the discussion | 9 | 7 |  |  |  |
| Express my views freely | 11 | 5 |  |  |  |
| Feel confident that my input will be used by the research team | 9 | 6 | 1 |  |  |
| Provided with proper supports to join the workshop | 11 | 5 |  |  |  |
| Good use of my time | 10 | 6 |  |  |  |
| Enjoyed the workshop | 10 | 6 |  |  |  |

1. Questionnaire wording has been abbreviated for clarity and conciseness; Response formats are represented as +++ for strongly agree, ++ for agree, +/- for neutral, - - for disagree, and - - - for strongly disagree

2. Not all attendees of the workshop completed the evaluation survey

## **3. Feedback from Family Caregiver Advisory Group**

| **Administered scale - Time in project (number of feedback received)** | **+++** | **++** | **+/–** | **– –** | **– – –** |
| --- | --- | --- | --- | --- | --- |
| **Modified PPEET – After Meeting #1 (Onboarding)** Response rate 75% (n = 3) | | | | | |
| Easy to attend | 3 |  |  |  |  |
| Clear and understandable | 2 | 1 |  |  |  |
| Helped me understand the study and why it was being done | 3 |  |  |  |  |
| Helpful in preparing me for the Delphi study | 2 | 1 |  |  |  |
| Better informed about my role in the project | 2 | 1 |  |  |  |
| Length of the session was acceptable | 2 | 1 |  |  |  |
| Provided proper supports to join (e.g., time of the session, technical support) | 3 |  |  |  |  |
| Enjoyed the onboarding session | 3 |  |  |  |  |
| Good use of my time | 3 |  |  |  |  |
| Satisfied with the session | 3 |  |  |  |  |
| **Modified PPEET – After Meeting #2 (Delphi Feedback)** Response rate 100% (n = 5) | | | | | |
| Easy to attend | 2 | 2 | 1 |  |  |
| Clear and understandable | 4 | 1 |  |  |  |
| Understood the purpose of the meeting | 5 |  |  |  |  |
| Express my views freely | 4 | 1 |  |  |  |
| Feel confident that my input will be used by the research team | 3 | 2 |  |  |  |
| Better informed about the Delphi study | 3 | 2 |  |  |  |
| Acceptable meeting length | 4 | 1 |  |  |  |
| Provided proper supports to join (e.g., time of the session, technical support) | 4 | 1 |  |  |  |
| Good use of my time | 4 | 1 |  |  |  |
| Satisfied with the meeting | 4 | 1 |  |  |  |
| **Modified PPEET – After Meeting #3 (Consensus Meeting and E&E Preparatory Meeting)** Response rate 75% (n = 3) | | | | | |
| Easy to attend | 1 | 2 |  |  |  |
| Clear and understandable | 1 | 2 |  |  |  |
| Understood the purpose of the meeting | 3 |  |  |  |  |
| Express my views freely | 1 | 2 |  |  |  |
| Better informed about the Delphi study | 2 | 1 |  |  |  |
| Acceptable meeting length | 2 | 1 |  |  |  |
| Provided proper supports to join (e.g., time of the session, technical support) | 3 |  |  |  |  |
| Good use of my time | 3 |  |  |  |  |
| Satisfied with the meeting | 3 |  |  |  |  |
| **Modified PPEET & PIERS – After Meeting #4 (Knowledge Translation, Reflections, TIDieR-C Info)** | | | | | |
| *Modified PPEET – Feedback on FCAG Meeting; Response rate 80% (n = 4)* | | | | | |
| Easy to attend | 3 | 1 |  |  |  |
| Provided proper supports to join (e.g., time of the session, technical support) | 3 | 1 |  |  |  |
| Clear and understandable | 3 | 1 |  |  |  |
| Understood the purpose of the meeting | 3 | 1 |  |  |  |
| Enough information to contribute to discussion | 3 | 1 |  |  |  |
| Express my views freely | 3 | 1 |  |  |  |
| Feel confident that my input will be used by the research team | 2 | 2 |  |  |  |
| Good use of my time | 3 | 1 |  |  |  |
| Satisfied with the meeting | 3 | 1 |  |  |  |
| **Modified PEIRS –** *Feedback on overall experience with SPIRIT \| CONSORT-C Response rate 80% (n = 4)* | | | | | |
| Understood the objective(s) of the project | 2 | 2 |  |  |  |
| Sufficient support to contribute to the project | 1 | 3 |  |  |  |
| Understood how to contribute to the project | 2 | 2 |  |  |  |
| Communication with research team clear | 3 | 1 |  |  |  |
| Being part of the Family Caregiver Advisory Group was worth my time | 3 | 1 |  |  |  |
| Contributed by providing my perspective | 1 | 3 |  |  |  |
| Any concerns were addressed | 2 | 2 |  |  |  |
| Research team was open to receiving my views | 2 | 2 |  |  |  |
| Offered sufficient recognition | 3 | 1 |  |  |  |
| Enjoyed being part of the Family Caregiver Advisory Group | 3 | 1 |  |  |  |
| **Modified PPEET –** *Feedback on TIDieR-C Information Meeting Response rate 75% (n = 3)** | | | | | |
| Easy to attend | 2 | 1 |  |  |  |
| Provided proper supports to join (e.g., time of the session, technical support) | 2 | 1 |  |  |  |
| Clear and understandable | 2 | 1 |  |  |  |
| Understood the purpose of the meeting | 2 | 1 |  |  |  |
| Enough information to contribute to discussion | 2 | 1 |  |  |  |
| Express my views freely | 2 | 1 |  |  |  |
| Good use of my time | 2 | 1 |  |  |  |

Questionnaire wording has been abbreviated for clarity and conciseness; Response formats are represented as +++ for strongly agree, ++ for agree, +/- for neutral, - - for disagree, and - - - for strongly disagree

*One family caregiver was unable stay for the TIDieR-C information meeting, and so the denominator is 4 instead of 5.

## **4. Feedback from Delphi Panellists**

| **Administered scale - Time in project (number of feedback received)** | **+++** | **++** | **+/–** | **– –** | **– – –** |
| --- | --- | --- | --- | --- | --- |
| **Modified PPEET – After Delphi Training Session;** Response rate 93% (n = 13) | | | | | |
| Clear and understandable | 8 | 4 |  |  | 1 |
| Clear understanding of the training session’s purpose | 5 | 6 |  | 1 | 1 |
| Helped me understand project purpose and goals | 9 | 3 |  |  | 1 |
| Helped prepare me for the Delphi study | 8 | 3 | 1 |  | 1 |
| Better informed about my role as a Delphi panellist | 9 | 2 | 1 |  | 1 |
| Length of the session was acceptable | 9 | 3 |  |  | 1 |
| Provided proper supports to join (e.g., time of the session, technical support) | 7 | 4 | 1 |  | 1 |
| Good use of my time | 10 | 1 | 1 |  | 1 |
| Enjoyed the training session | 6 | 5 | 1 |  | 1 |
| **Modified Acceptability E-Scale – After Delphi Study**; Response rate 100% (n = 14) | | | | | |
| Ease of completing Delphi study | 2 | 7 | 4 | 1 |  |
| Understandability of content in Delphi surveys | 2 | 8 | 3 | 1 |  |
| Enjoyed completing the Delphi surveys | 5 | 7 | 2 |  |  |
| Could share thoughts and opinions | 7 | 4 | 3 |  |  |
| Acceptable amount of time in completing the surveys | 5 | 4 | 5 |  |  |
| Overall satisfied with Delphi surveys | 4 | 9 | 1 |  |  |
| Training session prior to Delphi study was helpful | 7 | 4 | 2 | 1 |  |
| **Modified PEIRS – After Delphi Study;** Response rate 100% (n = 14) | | | | | |
| Introduced to members of the research team | 6 | 7 | 1 |  |  |
| Understood objective(s) of the Delphi study | 6 | 7 | 1 |  |  |
| Understood how to contribute to Delphi study | 7 | 7 |  |  |  |
| Received sufficient explanation about the Delphi study | 6 | 7 | 1 |  |  |
| Able to complete the Delphi study | 9 | 5 |  |  |  |
| Clear communication with research team | 7 | 7 |  |  |  |
| Worthwhile of my time | 6 | 8 |  |  |  |
| Sufficient time to complete tasks | 6 | 7 | 1 |  |  |
| Contributed my perspectives | 5 | 8 | 1 |  |  |
| Manageable workload | 5 | 9 |  |  |  |
| Sufficient support to contribute | 6 | 6 | 2 |  |  |
| Concerns were addressed | 6 | 7 | 1 |  |  |
| Research team was open to my views | 10 | 4 |  |  |  |
| Sufficient recognition for my contributions | 8 | 5 | 1 |  |  |
| Enjoyed completing the Delphi study | 7 | 6 | 1 |  |  |

Questionnaire wording has been abbreviated for clarity and conciseness; Response formats are represented as +++ for strongly agree, ++ for agree, +/- for neutral, - - for disagree, and - - - for strongly disagree

## **5. Feedback from Consensus Meeting Attendees (Family Advisors)**

| **Administered scale - Time in project (number of feedback received)** | **+++** | **++** | **+/–** | **– –** | **– – –** |
| --- | --- | --- | --- | --- | --- |
| **Modified Acceptability E-Scale – After Consensus Meeting**; Response rate 100% (n = 4) | | | | | |
| Ease of attending Consensus Meeting | 1 | 2 | 1 |  |  |
| Understandability of content at Consensus Meeting | 2 | 1 | 1 |  |  |
| Enjoyed attending Consensus Meeting | 2 | 1 | 1 |  |  |
| Helpful for sharing thoughts and opinions | 2 | 2 |  |  |  |
| Acceptable amount of time to attend the Consensus Meeting | 2 | 1 | 1 |  |  |
| Overall satisfied with Consensus Meeting | 2 | 2 |  |  |  |
| Training session prior to Consensus Meeting was helpful | 2 | 2 |  |  |  |
| **Modified PEIRS – After Consensus Meeting**; Response rate 100% (n = 4) | | | | | |
| Understood objective(s) of the Consensus Meeting | 3 | 1 |  |  |  |
| Understood how to contribute to Consensus Meeting | 2 | 2 |  |  |  |
| Received sufficient explanation about the Consensus Meeting | 3 | 1 |  |  |  |
| Attending the Consensus Meeting was worth my time | 3 | 1 |  |  |  |
| Contributed my perspectives |  | 4 |  |  |  |
| Manageable workload prior to the Consensus Meeting | 2 | 2 |  |  |  |
| Sufficient support to contribute | 4 |  |  |  |  |
| Concerns were addressed | 3 | 1 |  |  |  |
| Research team was open to my views | 3 | 1 |  |  |  |
| Sufficient recognition for my contributions | 4 |  |  |  |  |
| Enjoyed being part of the Consensus Meeting | 4 |  |  |  |  |
| **Perceived Impact – After Consensus Meeting**; Response rate 100% (n = 4) | | | | | |
| Able to contribute to the Consensus Meeting | 1 | 3 |  |  |  |
| Comfortable in communicating views | 1 | 3 |  |  |  |
| Input provided will make a difference in reporting guideline development | 1 | 3 |  |  |  |

Questionnaire wording has been abbreviated for clarity and conciseness; Response formats are represented as +++ for strongly agree, ++ for agree, +/- for neutral, - - for disagree, and - - - for strongly disagree

## **6. Feedback after Explanation and Elaboration (E&E) Writing Process**

| **Administered scale - Time in project (number of feedback received)** | **+++** | **++** | **+/–** | **– –** | **– – –** |
| --- | --- | --- | --- | --- | --- |
| **Modified Acceptability E-Scale – After E&E Writing Process;** Response rate 75% (n = 3) | | | | | |
| Ease of contributing to the E&E documents |  |  | 2 | 1 |  |
| Understandability of process in contributing to the E&E documents |  | 2 | 1 |  |  |
| Enjoyed being part of the E&E writing team | 1 | 1 | 1 |  |  |
| Acceptable amount of time to contribute to the E&E documents | 1 |  | 1 | 1 |  |
| Overall satisfied with being part of the E&E writing team |  | 1 | 2 |  |  |
| Training session prior to Consensus Meeting was helpful | 2 | 1 |  |  |  |
| **Modified PEIRS – After E&E Writing Process;** Response rate 75% (n = 3) | | | | | |
| Understood objective(s) of the E&E documents | 3 |  |  |  |  |
| Understood how to contribute to E&E documents |  | 3 |  |  |  |
| Received sufficient explanation on how to contribute to the E&E documents | 1 | 1 | 1 |  |  |
| Being part of the E&E writing team was worth my time | 2 | 1 |  |  |  |
| Contributed my perspectives |  | 2 | 1 |  |  |
| Manageable workload being part of the E&E writing team | 1 |  | 1 | 1 |  |
| Sufficient support to contribute |  | 1 | 2 |  |  |
| Concerns were addressed | 1 |  | 2 |  |  |
| Fellow writing team member(s) were open to receiving my views | 1 | 1 | 1 |  |  |
| Sufficient recognition for my contributions | 2 |  | 1 |  |  |
| Enjoyed being part of the E&E writing team | 2 |  | 1 |  |  |
| **Perceived Impact – After E&E Writing Process;** Response rate 75% (n = 3) | | | | | |
| Able to contribute to the E&E documents | 1 | 2 |  |  |  |
| Comfortable in contributing to the E&E documents |  | 2 | 1 |  |  |
| Input provided will make a difference in reporting guideline development | 2 | 1 |  |  |  |

Questionnaire wording has been abbreviated for clarity and conciseness; Response formats are represented as +++ for strongly agree, ++ for agree, +/- for neutral, - - for disagree, and - - - for strongly disagree

# **F. eTables and eFigure**

## **eTable 1. Twenty-one applications of 17 “Blueprint” recommendations (3) in the development of SPIRIT | CONSORT-Children & Adolescents 2025**

| **Recommendation(3)** | **Application** |
| --- | --- |
| **Project Launch** | |
| 1. *Include experienced patient partner(s) as member(s) of the steering committee:* Invite the patient partner(s) to design and (co-) lead the PPI component to mitigate power imbalances and ensure relevance of project to the end-users. | 1. A dedicated patient engagement expert (MS) was part of the core project team and co-led the design and execution of the youth and family caregiver involvement strategy. |
| 1. *Plan the PPI component:*   Decide on:  (a) the number of patient and public members to involve,  (b) how to recruit them,  (c) when and how to involve them in the project, and  (d) how to evaluate the PPI component of the project with both patients and public members, and with the research team. | 1. (a) We planned to form a Youth Advisory Group (YAG) with six youth ages 12-19 years and a Family Caregiver Advisory Group (FCAG) of five parents and guardians with relevant lived experiences. For the Delphi study, we planned to recruit a maximum of 10 family caregivers and 5 young people (ages 19-24 years) to be Delphi panellists.   (b) We identified potential advisors and panellists through various advisory groups and patient groups across Canada. For Young Person Reporting Guideline (YPRG) workshops, we connected with the youth facilitator experts from YPAGs from four countries: England, France, Spain, and Scotland, with the aim of each group having at least 10 youth who can attend the workshops with their own YPAG. Youth across Canada were also identified through various existing advisory groups and patient groups.  (c) We offered various ways to get involved based on each person’s desired level of commitment, with options to be involved throughout the project or just in select stages of the project (e.g., 2. Delphi study).  (d) We collected feedback after each meeting or project stage. Evaluation surveys with modified Public and Patient Engagement Evaluation Tool (PPEET) questions were prepped for after each advisory group meeting and training sessions (e.g., onboarding, Delphi training, Consensus Meeting prep). After certain project stages (2. Delphi study, 3. Consensus Meeting), the evaluation surveys contained modified questions from the Acceptability E-Scale,(7) Patient Engagement in Research Scale (PEIRS) (8), and additional questions related to impact. At times, qualitative feedback was also obtained through debrief focus group sessions (i.e., post Delphi study, end of the project). After the Consensus Meeting, all other attendees were also invited to complete a short survey to evaluate the PPI component, and their impact, on the Consensus Meeting’s process and results. |
| 1. *Allocate budget for compensation:* Compensate patients and public members and the patient partner(s) for their time and contributions to various project stages in accordance with (inter)national guidelines, factoring in preparation time. | 1. We budgeted for compensation for all young people and family caregivers. Compensation figures were based on the Canadian SPOR guidelines: $25/hour in 2023 (9, 10), which was increased to $40/hour in 2024. Due to regulations in different jurisdictions, young people who joined the YPRG workshops in countries outside of Canada were compensated according to guidelines set forth by their own group. 2. We outlined the compensation amount that young people and family caregivers would receive for each project stage in writing and during meetings so all young people and family caregivers had clear expectations on what they can expect to receive for their time and contributions |
| **Delphi Study Preparation** | |
| 1. *Deliver an onboarding session:* Review the project objectives and methods, essential concepts, why PPI is important, and be clear on the roles and expectations of patients and public members, and what patients and public members can expect in return. Ask the project team to join the first 10 minutes to introduce themselves and get to know the patients and public members. Allow time for questions during and after the session. | 1. We invited all potential youth advisors and family caregiver advisors to an onboarding session (1 hour and 1.5 hours, respectively) to go over project background and rationale, important concepts, importance of their involvement, roles, expectations, and compensation. The principal investigator (MO) was present throughout the onboarding sessions and was available to answer any questions from attendees. Time was allotted throughout the session and at the end for any questions and discussion. Similarly, a training session (60 minutes) was conducted with all potential Delphi panellists. 2. A preparatory meeting prior to the Consensus Meeting was conducted with family caregiver advisors who would be attending. At this preparatory meeting, we went over what to expect, how to contribute effectively, their roles, and any questions they may have. The subsequent Explanation and Elaboration (E&E) documents writing process (Stage 4) was also introduced as an optional opportunity for anyone who was interested in writing/reviewing. |
| 1. *Draft documents pertaining to the background of the reporting item and/or glossaries of concepts:* Pilot materials with patient partner(s); include background documents and glossaries in the Delphi survey; update this information after each Delphi round. | 1. We obtained feedback from the youth advisors and family caregiver advisors for informational material (e.g., flyer, information sheet) that was used to recruit youth for the YPRG workshops (Stage 1) and family caregivers and young people (ages 19-24) for the Delphi study (Stage 2). The Glossary for the Delphi study was reviewed by a family caregiver advisor. |
| 1. *Pilot the Delphi survey with patient partner(s):* Ask patient partner(s) to test Delphi surveys to ensure clarity on the objectives, methods, instructions, and wording. | 1. We reached out to family caregiver advisors prior to the launch of Rounds 1 and 2 of the Delphi survey, which were the lengthiest. Family caregiver advisors were asked if they would be interested in pilot testing and providing feedback, which was presented as an optional activity. While piloting for the first Delphi round was for the full survey, we prepared a mini truncated version of the Round 2 for piloting purposes to obtain targeted feedback and to reduce the time needed to pilot. Round 3 was not piloted as it was significantly shorter, and the format was very similar to Round 2. All pilot testers were compensated for their time, according to the communicated standard. |
| **Delphi Study** | |
| 1. *Set a timeframe:* Estimate the duration to complete the Delphi survey, but state that it may take longer or shorter; highlight the possibility of completing the survey progressively in more than one sitting. | 1. We provided an estimated duration to complete the Delphi survey (e.g., “approximately 1 hour to complete”) during the Delphi training session and in the instructions for each round and expressed that it may take longer. We also offered all panellists, not just family caregivers and young people, the option to complete each Delphi round in more than one sitting through a “Save and Return” function. |
| 1. *Offer the option not to vote:* Include a ‘not my expertise’ option and emphasize in the instructions that it is okay to select this option. | 1. Instead of “not my expertise”, we received feedback from family caregiver advisors during an advisory group meeting that they would prefer this option be reworded to “I’m opting out”. As a result, “I’m opting out” was offered for all voting items in all Delphi rounds. We explained different possible reasons that someone may select this response (e.g., item outside of knowledge/expertise, prefer not to vote on the item) during the Delphi training session and in the survey instructions, and emphasized that it is okay to select this response. This option was available to all Delphi panellists. |
| 1. *Analyze and present responses of patients and public members as a separate stakeholder group:* Highlight responses of patients and public members if different from those of other stakeholders. | 1. We analysed the responses to all Delphi rounds separately for each panellist group. Additionally, we highlighted the responses of family caregivers and young people in the summary report after each round and on each voting page for the reporting items and during the Consensus Meeting (Stage 3). For *Youth Generated* and *Youth Endorsed* items, these items were clearly flagged as such in all Delphi rounds and at the Consensus Meeting. |
| **Between Delphi and Project Meetings** | |
| 1. *Follow up with patients and public members:* Besides planned evaluation methods, ask patients and public members if they have additional reflections on their involvement, and how they want to share their feedback. | 1. We implemented an open communication policy where family caregivers and young people were encouraged to reach out to the project manager (AB) with any questions or suggestions through e-mail. After Delphi Round 1, we offered family caregivers and young people who were panellists the opportunity to join a post-Delphi check-in session. Additionally, after the Delphi study was completed, all family caregiver and young people panellists were invited to attend a dedicated *post-Delphi debrief session* to reflect on their experiences completing the Delphi study. |
| 1. *Identify subsequent activities where patient and public input is desired:* Be flexible and offer patients and public members opportunities to engage in subsequent project activities, even if PPI in subsequent stages was not planned for from the start; establish what is needed for meaningful involvement. | 1. We offered youth advisors the opportunity to stay involved after the final Youth Advisory Group meeting and contribute to the development of knowledge translation deliverables, such as a video, to communicate to other young people and families on how the SPIRIT \| CONSORT-C guidelines can be used by youth. As the development of these supportive materials would take place after the final Youth Advisory Group meeting and after the finalization of the guidelines, we maintained flexibility on who would be involved in the development of these deliverables based on who was interested and available. |
| 1. *Inform patients and public members of their impact on the project:* Report back on how the project has changed or developed because of their contributions. | 1. We prepared “What We Heard and What We Did” documents and slides to report back to advisors on how their feedback impacted various materials (i.e., information sheet, flyer). We also did this for the YPRG workshops to share with youth workshop attendees how their original ideas became candidate reporting items. Additionally, changes could also be seen in deliverables (e.g., Delphi survey instructions, reporting items). |
| **Throughout the project** | |
| 1. *Send detailed ‘pre-work’ and information:* Ensure patient partner(s) co-writes or reviews all information and documents that will be sent to patients and public members. | 1. We developed materials (advisory group meeting slides, information sheets, evaluation surveys) with input from the patient engagement expert and obtained feedback from advisors where relevant (detailed under #5, above). 2. Prior to the Consensus Meeting, all attendees, including family advisors, were provided with the Pre-Consensus Meeting Package. |
| 1. *Remain flexible in methods and procedures:* Listen to the needs and wishes of patients and public members; remain flexible and willing to adjust methods and procedures as the project evolves. | 1. We originally offered family caregivers and young people the option to select one gift card out of a selection of five after each project stage that they were involved in. One family caregiver Delphi panellist suggested the use of the Canadian *EverythingCard* for compensation, which the team adopted to provide all family caregivers and young people more choice and flexibility in their gift card selections. The switch to *EverythingCard* streamlined and improved the efficiency of the compensation process, as the intermediary step of asking everyone for their gift card choice after a project stage was eliminated. |
| 1. *Consider it a mutual learning experience:* Create an atmosphere of reciprocity, encouraging researchers and patients and public members to learn from one another, and offer feedback at each project stage, underscoring that all contributions are valuable. | 1. We created multiple opportunities for family caregivers and young people to provide feedback through different mediums (detailed under #2d), which we discussed with them and let our project researchers learn from. Family caregivers and young people seized the opportunity to learn more about reporting guidelines, randomised controlled trials, and hone professional skills (e.g., writing, as part of the E&E team). 2. We learned from the feedback and experiences shared by the family caregivers and young people after every stage of the project. We learned what was effective and valued by patient and public partners (e.g., onboarding/training sessions, reliable point of contact, glossary) and what could be improved (e.g., providing clear exemplars to model after, more examples provided during training to illustrate concepts and methods, having more check-in sessions). |
| 1. *Prospectively capture the impact of PPI at each stage:* Outline in a separate record the tangible and measurable changes stemming from PPI input during each stage of the project; disseminate/publish these changes in a knowledge translation piece. | 1. We prospectively recorded the impact of family caregivers and young people on the development of SPIRIT \| CONSORT-C and summarized the impact at each stage (Table 1). |
| **Reporting the project** | |
| 1. *Use specific reporting standards to report on PPI:* Adhere to the GRIPP2 (11) reporting guideline to transparently and comprehensively account for PPI. | 1. For this manuscript, we reported the involvement of family caregivers and young people using the Guidance for Reporting Involvement of Patients and the Public (GRIPP2) reporting guideline (11). |

**Abbreviations**

SPIRIT: Standard Protocol Items: Recommendations for Interventional Trials

CONSORT: Consolidated Standards of Reporting Trials

YAG: Youth Advisory Group

YPAG: Young Person’s Advisory Groups

PPEET: Public and Patient Engagement Evaluation Tool

PEIRS: Patient Engagement in Research Scale

OSSU: Ontario SPOR SUPPORT Unit

SPOR: Strategy for Patient-Oriented Research

GRIPP2: Guidance for Reporting Involvement of Patients and the Public

## **eTable 2a. What worked well, with illustrative quotes from feedback received**

| **Worked well** | **Illustrative quotes** |
| --- | --- |
| **Team composition** | - Well, make sure you have an [point person] – because parents are gonna ask lots of questions, right? And like, I know for myself, kids in school. There's all that going on, you know, there's lots of layers right? So then sometimes you're so caught up in that. And then you're like, Okay, yes, I'm involved in this. and sometimes you feel like there's a gap. So you might go back and say, Hey, you just have questions right? So I think I would have for me, I just think, expect lots of questions and be patient. [*Regarding advice to researchers working on methodological reporting guidelines, during the post-Delphi debrief*] - Role of [point person] and [patient partner lead] – both are completely necessary in a project like this. I think I was in another meeting a week ago, and they were embarking on. They wanted to have people with lived experience to go through the Delphi process. And I had brought up, I said, Well, I just finished this thing, and you know there was a training session, and there's a you know, a parent person. And then there's the coordinator that you can ask questions. And they hadn't even thought of anything like that, and I just think I would have dropped out like a long time ago if those roles were not embedded into this project. [*Regarding advice to researchers working on methodological reporting guidelines, during the post-Delphi debrief*] - Having a point person was key. Having [point person] made it more personalized, and helped me to buy in and feel like I was making a meaningful contribution. The updates and prompts were perfect. I really felt heard and seen. [*Regarding advice to researchers working on methodological reporting guidelines, during the post-Delphi debrief*] - [Point person], [patient engagement expert] etc were all wonderful in anticipating our needs and providing what was needed. [*Regarding the consensus meeting*] - I really appreciated that [name redacted] told us she was there for support [*Regarding the consensus meeting*] |
| **Training and project materials** | **Training**   - From my point of view the [Delphi] training sessions was totally necessary. I would not have understood how to do the first survey without it. Before this exercise the word ‘Delphi’ was quite daunting for me. After the training, I had more confidence [*Regarding the Delphi training session*] - I appreciate the opportunity to connect with the various stakeholders, learning a little more about them each time increases my feeling of belonging to the group and the project. This enhances the fact that I also learn things and feel useful. The abilities of stakeholders to create a safe space to express questions and opinions. Information provided in advance. [*Regarding the Delphi training session*] - Well-laid out slides that clearly described the process, respectful discussion, lots of time for questions. The consensus meeting will be easier for us because of this pre-meeting. Lots of practical details were shared e.g. 1-2 sentences, not more than one minute, we only have ten minutes to discuss each item” [*Regarding the Pre-Consensus Meeting Prep*]   **Project materials**   - While it was **optional** to read slides beforehand, it helps some of us to think and be better prepared. [*Regarding the Post Delphi FCAG meeting #2*] - Very nice to have the slides to review beforehand; information was clear and concise. [*Regarding the Delphi training session*] - Typically this is where I mention that it is helpful to receive the slide deck ahead of the presentation so that I can form my questions ahead of time; however, the trainers provided the slide deck ahead of time! very happy about that as it made following along that much easier. [*Regarding the Delphi training session*]   **Glossary**   - I have been in many meetings where the scientists use all the terms very freely and I often feel intimidated. The glossary is helpful and I like how I can read it on my own time. [*Regarding the Delphi study*] - I liked having the glossary definitions right in the survey question (where applicable). [*Regarding the Delphi study*] - I also agree that it was helpful. I come from some research background. So a lot of the terms were terms I had known already… the terms were very clear, and the definitions as well [*Regarding the Delphi study*] |
| **Openness of research team** | - There is always room for improvement but the team is already inviting us to evaluate with good tools so it should continue like this. [*Regarding the Family Caregiver Advisor Onboarding Session*] - “It was really great to meet everyone and get to know them better. The informal environment allowed for openness and comfort in sharing. The team was very receptive to our feedback and ideas for change, this was really nice.” [*Regarding the* *Delphi feedback FCAG meeting #2*] - It is obvious that the feedback was taken seriously. Everybody seemed very comfortable to speak. [*Regarding the Delphi feedback FCAG meeting #2*] - Having the opportunity to give feedback via this session is great [*Regarding the Post Delphi debrief*] |

## **eTable 2b. Areas for improvement with illustrative quotes from feedback received**

| **Area for improvement*** | **Illustrative quotes** |
| --- | --- |
| **Training and examples** | - “I would’ve liked more training as well, and I like the idea above about visuals. I think more hands-on practice during the training (i.e. practice items) would have also been helpful” [*Regarding the* Post Delphi] - “Have the opportunity to set an example as a team. A practice” [*Regarding the E&E process*] - “have done Delphi's before but more examples would have been helpful” [*Regarding the Delphi study*] - “I think having a shared example here might have been beneficial. I went and looked at another E&E, but some may not have.” [*Regarding the E&E process*] |
| **Time** | - I would have liked to have a bit more time to look at the slides. [*Regarding the FCAG #2 meeting*] - Maybe add a little more time at the end for folks who want to stay on a linger a little but can 'end the meeting' for those who need to run. There might have been a bit too much material to cover in one hour but I appreciate [name redacted] trying to keep us mostly on time, lol! [*Regarding the* *FCAG #2 meeting*] - I found the time estimates to be a little short especially if you were to refer back to results from earlier rounds. [*Regarding the Delphi*] - The first survey was very long and I wasn’t quite prepared for that. Would have liked a bit more of a heads up. The other 2 went more smoothly because a) I was prepared b) I felt more confident [*Regarding the Delphi study*] - The original timeline was too short [*Regarding the E&E process*] - It was a lot more difficult than I expected. It also took more time than I realized. [*Regarding the E&E process*] |
| **Organized check-in meetings** | - “I would have valued a check in meeting for those having trouble and troubleshooting a bit together. I had a few emails but an optional group call with the leads would have been a great addition.” [*Regarding the E&E process*] - The process was well laid out overall. The most difficult part was not communicating effectively in team. The process could have benefited from a request to meet as a writing team prior to starting the writing process so we could understand strengths and plan together. [*Regarding the E&E process*] |

## **eFigure 1: Blueprint recommendations for involving patient and public partners in reporting guideline development (3)**


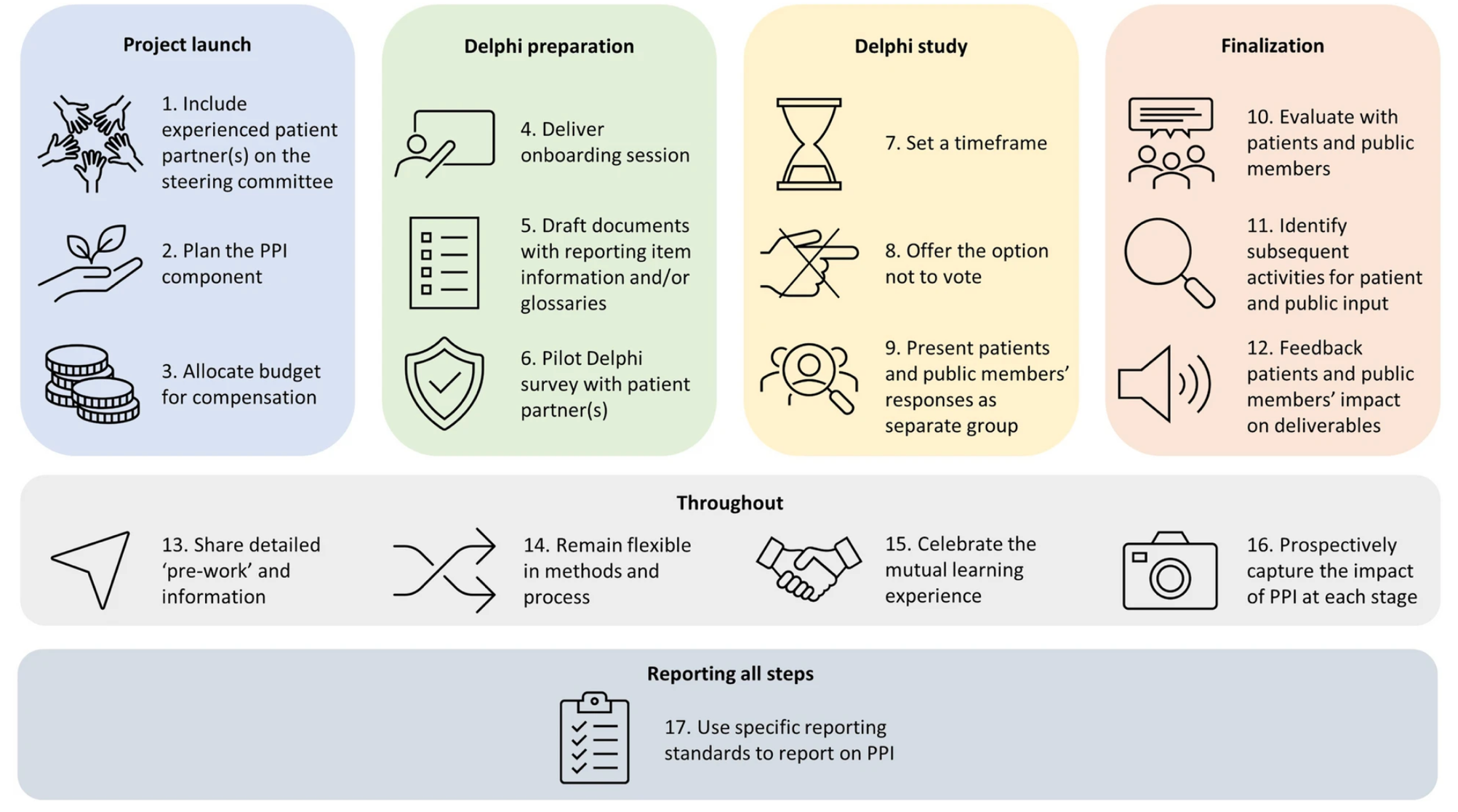
Used with permission in accordance with CC BY 4.0 guidelines

## **References**

1. Baba A, Smith M, Potter B, Chan A-W, Moher D, Toulany A, et al. Enhancing the reporting and impact of paediatric randomised trials: CONSORT-Children and Adolescents (CONSORT-C) 2025 extension. Under review.

2. Baba A, Smith M, Potter B, Chan A-W, Moher D, Toulany A, et al. Enhancing the reporting and usefulness of paediatric randomised trial protocols: SPIRIT-Children and Adolescents (SPIRIT-C) 2025 extension. Under review.

3. Elsman EBM, Smith M, Hofstetter C, Gavin F, Jobson E, Markham S, et al. A blueprint for patient and public involvement in the development of a reporting guideline for systematic reviews of outcome measurement instruments: PRISMA-COSMIN for OMIs 2024. Res Involv Engagem. 2024;10(1):33.

4. Baba A, Smith M, Potter B, Chan A-W, Moher D, Toulany A, et al. SPIRIT-C 2025 Explanation and Elaboration: Recommendations for enhancing the reporting and usefulness of paediatric randomised trial protocols. Under review.

5. Baba A, Smith M, Potter B, Chan A-W, Moher D, Toulany A, et al. CONSORT-C 2025 Explanation and Elaboration: Recommendations for enhancing the reporting and impact of paediatric randomised trials. Under review.

6. Goren K, Lai V, Baba A, Smith M, Hoffmann T, Offringa M. Enhanced transparency and precision in the reporting of interventions in child health clinical trial reports using the Template for the Intervention Description and Replication (TIDieR) checklist: TIDieR-Children and Adolescents. In preparation.

7. Tariman JD, Berry DL, Halpenny B, Wolpin S, Schepp K. Validation and testing of the Acceptability E-scale for web-based patient-reported outcomes in cancer care. Appl Nurs Res. 2011;24(1):53-8.

8. Hamilton CB, Hoens AM, McQuitty S, McKinnon AM, English K, Backman CL, et al. Development and pre-testing of the Patient Engagement In Research Scale (PEIRS) to assess the quality of engagement from a patient perspective. PLoS One. 2018;13(11):e0206588.

9. SPOR Networks in Chronic Diseases and the PICHI Network. Recommendations on Patient Engagement Compensation 2018 [Available from: <https://diabetesaction.ca/wp-content/uploads/2018/07/TASK-FORCE-IN-PATIENT-ENGAGEMENT-COMPENSATION-REPORT_FINAL-1.pdf>. .

10. Alliance SE. Patient and Public Partner Appreciation Policy and Protocol 2022 [Available from: <https://sporevidencealliance.ca/wp-content/uploads/2022/01/SPOREA_Patient-and-Public-Appreciation-Policy_2021.01.14-1.pdf>.

11. Staniszewska S, Brett J, Simera I, Seers K, Mockford C, Goodlad S, et al. GRIPP2 reporting checklists: Tools to improve reporting of patient and public involvement in research. BMJ. 2017;358:j3453.
